# A three evolutionary game model for driving mechanism of industry-university-research collaborative innovation in agricultural innovation ecosystems

**DOI:** 10.1371/journal.pone.0289408

**Published:** 2023-08-03

**Authors:** Xiaona Hou, Xiangxiao Gao, Shi Yin, Jianmin Li

**Affiliations:** 1 School of Accounting, Hebei Finance University, Baoding, China; 2 College of Economics and Management, Hebei Agricultural University, Baoding, China; East China Normal University, CHINA

## Abstract

Based on the current state of China’s agricultural industry, this article proposes an integrated framework for the agricultural innovation ecosystem in developing countries. Furthermore, a dynamic simulation model is constructed to analyze the game process and factors influencing the agricultural innovation ecosystem. The results indicate that industry-university-research collaboration serves as the main source of innovation within the agricultural innovation ecosystem, playing a central role in its development. The willingness to participate, cost of participation, and establishment of default fees by governments, agro-related enterprises, universities, and research institutions have significant implications for the evolution of collaborative innovation within agricultural innovation ecosystems. In order to promote the evolution of the system, agriculture-related enterprises, universities, and research institutions should establish more effective reward and punishment mechanisms, as well as cost control mechanisms. Governments should set reasonable regulatory costs and incentive intervals to actively foster a collaborative innovation atmosphere. The innovation points of this paper are as follows: extending the theory of innovation ecosystems to agriculture, particularly in developing countries characterized by imbalanced and insufficient development. A game model is also constructed to represent the collaborative innovation evolution among government, agriculture-related enterprises, universities, and research institutions, with the government as the endogenous variable. Through numerical simulation, the dynamic evolution process of collaborative innovation within the agricultural innovation ecosystem is revealed. This research enriches and expands upon innovation ecosystem theory, providing guidance for the of innovation ecology in agriculture through mathematical models in developing countries. This, in turn, promotes the convergence of symbiotic, shared, and-creation development.

## 1. Introduction

Rural revitalization is a complex and systematic project, related to building China into a modern socialist country in a comprehensive manner [1]. In implementing the rural revitalization strategy, high-quality agricultural development is the foundation, innovation-driven development is the core driving force, and integrated urban and rural development is considered an effective path. The Fifth Plenary Session of the 19th CPC Central Committee emphasized the central role of innovation in the construction of agricultural modernization, which requires the continued strengthening of national strategic scientific and technological forces and the self-sufficiency and self-improvement of science and technology as a strategic support for national development [2]. The 14^th^ Five-Year Plan further consolidates the core position of innovation, emphasizing innovation-driven, high-quality supply-led and new demand creation [3], interpreting the meaning of innovation from a new aspect and regarding innovation as the fundamental driving force leading the development priority of agriculture and rural areas. The No.1 central document for 2022 put forward the basic principles of ensuring stability and making progress while maintaining stability. The stability of the basic agricultural sector must be preserved, while continuing to promote rural revitalization. Innovations in the modern seed industry, agricultural equipment, digital agriculture, and agricultural and rural reform are fundamental driving forces for high-quality, sustainable, and stable agricultural development. The introduction of relevant policy documents has further pointed out the direction for the development of agricultural modernization, and the leading role of innovation for rural revitalization must be brought into play, so as to realize a convergent development path of symbiosis, creation and sharing [4]. Agricultural innovation should also follow the concept of integrated development through symbiosis, innovation, and sharing, thus promoting the coordination and innovation of multiple entities. From the perspective of agricultural innovation practice, there are still many problems in the processes of agricultural production and circulation, such as the mismatch between production mode and technology allocation [5], immature enterprise management systems [6], weak multi-subject coordination mechanisms [7], and low conversion rate of agricultural scientific and technological achievements [8]. In the final analysis, these issues are due to the government, agriculture-related enterprises, universities, research institutions, and other agents not having established an effective coordination mechanism, resulting in difficulties in forming long-term stable cooperative relationships. Modern innovation is no longer limited to technological innovation, enterprise innovation, and industrial innovation, but emphasizes the construction of interactions between the innovation subject and innovation environment, closely related to the concept of an innovation ecosystem [9]. By introducing ecological theory into the process of agricultural innovation, these dynamic, complex, and systematic problems can be, in essence, solved. According to ecological theory, multiple subjects can give play to their niche advantages, find the space they need to survive, integrate and utilize heterogeneous innovation resources; in this case, in order to form a good agricultural innovation ecosystem and promote the improvement of agricultural innovation ability.

Compared with other industries, most agriculture-related enterprises are small- and medium-sized enterprises, which find it difficult to obtain the key technologies and capabilities required for innovation solely by their own means. Agricultural intellectual property rights are difficult to protect, and successful innovative technologies and products are easy to imitate, and because of the short-term unpredictability of basic research, agribusinesses have little incentive to invest independently in innovation [10]. Instead, the government must play a role in guiding universities and research institutions to establish industry–university–research cooperation based on market demand, in order to effectively promote the development of agricultural innovation. Industry-university-research synergistic innovation, as one of the most dynamic and competitive innovation models [11], can promote vertical and horizontal integration of agricultural innovation ecosystems based on a more open innovation perspective. Vertically the collaborative innovation of industry-university-research can open the five major links of agricultural products production, processing, storage, transportation and marketing, solve the synergistic problems of each link, and establish the connecting channels of one, two and three industries, form the synergistic mechanism within and between industries, and promote the deep integration of one, two and three industries. Horizontally, the innovation results outputted through the digital cooperation of industry, university and research can improve social and economic benefits, attract more diversified and wider innovation subjects to join the system, and achieve win-win situation for multiple parties [12]. Industry-university-research Collaboration can promote collaborative cooperation among innovation subjects, which can realize resource sharing and complementary advantages, effectively disperse risks and reduce transaction costs, enable each party to meet its own value pursuit, and promote the achievement of value consensus within the agricultural innovation ecosystem. Under the role of common value identity, the interaction activities related to collaborative innovation between innovation subjects around industry-university-research will increase, and through continuous interaction and feedback more relationships, resources and networks will be integrated to reconstruct new ways of value creation and realize the virtuous cycle operation of agricultural innovation ecosystem under the guidance of value co-creation [13]. It can be seen that industry–university–research collaborative innovation and value co-creation are the core characteristics that distinguish the agricultural innovation ecosystem from other ecosystems [14].The collaborative efforts of industry, academia and research, which are integrated in the common demand of value co-creation, will promote the sustainable and dynamic evolution of the agricultural innovation ecosystem [15]. Therefore, expanding the boundaries of industry-university-research collaborative innovation organizations, building an open and dynamic agricultural innovation ecosystem, and increasing the frequency of interaction among innovation subjects and the addition of new elements will essentially enhance the agricultural independent innovation capacity. What is an agricultural innovation ecosystem, after all? What are the components of an agricultural innovation ecosystem? How can the dynamic evolution of agricultural innovation ecosystem through synergistic innovation? Solving these problems will help integrate agricultural innovation resources, promote horizontal and vertical integration of the agricultural industry from a more open perspective, and promote the evolution of the agricultural innovation ecosystem to a higher order. In view of the above, in this paper, we provide an in-depth study of the previous relevant literature and evolutionary game theory, involving aspects such as ecology and self-organization, as well as the definition of the agricultural innovation ecosystem concept to analyze its constituent elements. On this basis, an evolutionary model is constructed to simulate the system of collaborative innovation and analyze the game in relation to the subject, in order to reveal the logical law of agricultural innovation ecosystem evolution. This provides intelligent support and decision-making reference for agricultural industry development to overcome barriers to transformation and upgrading.

The innovation points of this paper are as follows: Based on the research perspective of innovation ecosystem, which is at the forefront of innovation theory, this paper locates agriculture as a weak industry and explores the inner logic and mechanism of agricultural innovation ecosystem, and the related research expands the content and application scope of innovation ecosystem theory. In addition, compared to the existing literature that only introduces the government as an exogenous parameter variable in the game between industry–university–research, this paper considers the government as an important innovation subject to construct a three-party game model of collaborative innovation evolution among the government, agriculture-related enterprises, universities & research institutions, and reveals the dynamic evolution process of collaborative innovation in agricultural innovation ecosystem through numerical simulation, which provides a reference contribution for decision making to promote collaborative innovation in agricultural innovation ecosystem to achieve value co-creation.

The rest of this paper is as follows. Section 2 is the formulation of an agricultural innovation ecosystem. Methodology is shown in Section 3. Section 4 is numerical simulation analysis. Conclusions and future prospects are presented in Section 5.

## 2. Formulation of an agricultural innovation ecosystem

In the environment of globalization of agricultural economy, innovation has become the main way for developed countries and even developing countries to gain competitive advantage, but due to the limitation of innovation resources, developing countries often face multiple difficulties to enhance their independent innovation capacity [16]. The only way to solve the fundamental problem is to adopt open innovation. The innovation ecosystem theory provides an effective path for developing countries to overcome the low-end lock of agricultural science and technology innovation and improve the quality of agricultural development. As the largest developing country, China’s agricultural development is in an important period of shifting from a high growth stage to a high quality development stage, and a study of China’s agricultural innovation ecosystem can provide an important contribution to the ecological leap of agricultural innovation systems in developing countries. This paper takes Chinese agricultural industry as the research scope, and the study of agricultural innovation ecosystem can provide intellectual support and decision-making reference for the planning and deployment of China’s agricultural innovation-driven development, as well as provide experience in concepts, methods and paths for China to promote human life in the community and actively promote developing countries to break through the dilemma of autonomous agricultural innovation development.

### 2.1 Connotation and composition of agricultural innovation ecosystem

The innovation ecosystem is an extension of the business ecosystem [17], which was formally proposed by the U.S. President’s Council of Advisors on Science and Technology (PCAST) in 2004. Since then, the research on innovation ecosystems have become a hot topic in academic circles. At present, the definition of an innovation ecosystem is relatively loose and fragmented [18], and can be roughly divided into theoretical perspectives from systematics [19], factor flows [20], innovation networks [21], collaboration [22], responsible innovation [23], and so on. Scholars generally refer to the ideas of ecology and regard the innovation ecosystem as a dynamic and complex system formed by the interconnected and collaborative evolution of different stakeholders and the external environment [24]. With the deepening of research, the concept of an innovation ecosystem has been used more and more commonly to study the collaborative innovation of multiple actors in value co-creation [25]. Some scholars have stated that an innovation ecosystem is a collaborative innovation network centered on value co-creation [26]. Some other scholars believe that innovation ecosystem is an alliance structure formed through the cooperation of heterogeneous subjects, in order to realize core value propositions [27, 28].

As one of the important branches of innovation ecosystem, agricultural innovation ecosystem is an inevitable choice to deepen the reform of science and technology system, and an important support for the development of Chinese agricultural innovation. In recent years, scholars have begun to focus on the study of agricultural innovation ecosystems, which has laid the theoretical foundation for this paper. Pigford (2018) proposed an umbrella concept that defines agricultural innovation ecosystems as interdependent and nested interconnected innovation networks formed by multiple innovation players in pursuit of common value creation [29]. Wen (2019) considered agricultural innovation ecosystem as an innovation network composed of agro-involved firms, suppliers, consumers, and the external environment in which they were located, which was loose in nature [30]. Based on the architect theory, Li (2022) divided the agricultural innovation ecosystem into three parts: internal architects, external architects, and the environment, each of which interacts with each other to achieve resource integration and thus promote the value co-creation of the system [31]. Thus, on the one hand, it can be seen that agricultural innovation ecosystem contains some common elements of innovation ecosystem, and involves diversified innovation subjects such as agriculture-related enterprises, government, universities and research institutions [32]. In the whole process from technological research and development to commercialization, multiple innovation subjects are connected with each other, forming a network structure with multiple cycles, reciprocation, and cooperation of multiple subjects [33]. An agricultural innovation ecosystem aims to promote the improvement of agricultural innovation capacity, ultimately achieving the improvement of economic, social, and ecological benefits. In order to adapt to changes in the innovation environment, the innovation main body must facilitate the exchange of substances, energy, and information, thus creating a system with ecological and dynamic characteristics. Through cooperation, the realization of resource sharing, and complementary advantages, the value pursuits of various subjects can be satisfied, thus promoting internal value consensus of the system and, finally, realizing value co-creation. On the other hand, from the reality of China, there are still some limitations of innovation resources in the agricultural industry compared with other industries, leading to some special features of the agricultural innovation ecosystem. Agricultural innovation activities should take into account regional characteristics [34]; only the participation of government forces can ensure that innovation activities can be carried out smoothly [35]; it is necessary to rely on collaborative innovation between industry, academia and research to promote the improvement of independent innovation capacity [36]; special industries should be used as a grasp to highlight the competitive advantages of unique regional resources, make the regional brand bigger and stronger, and consolidate and strengthen the basic position of agriculture [12].

In summary, based on a large amount of literature, this paper defines the agricultural innovation ecosystem for developing countries from the actual Chinese agricultural industry as follows: Within the scope of a certain time and space, agricultural-related enterprises, universities, and research institutions (e.g., the multivariate innovation main body with industry–university–institution cooperation as the core), under the guidance of the government and community interactions, promote agriculture industry innovation and create an innovation environment which more effectively fosters material, energy, and information flows, leading to a symbiotic, creative, and co-prosperous common evolution of the system.

### 2.2 Integration framework for agricultural innovation ecosystem

**Fig 1:**
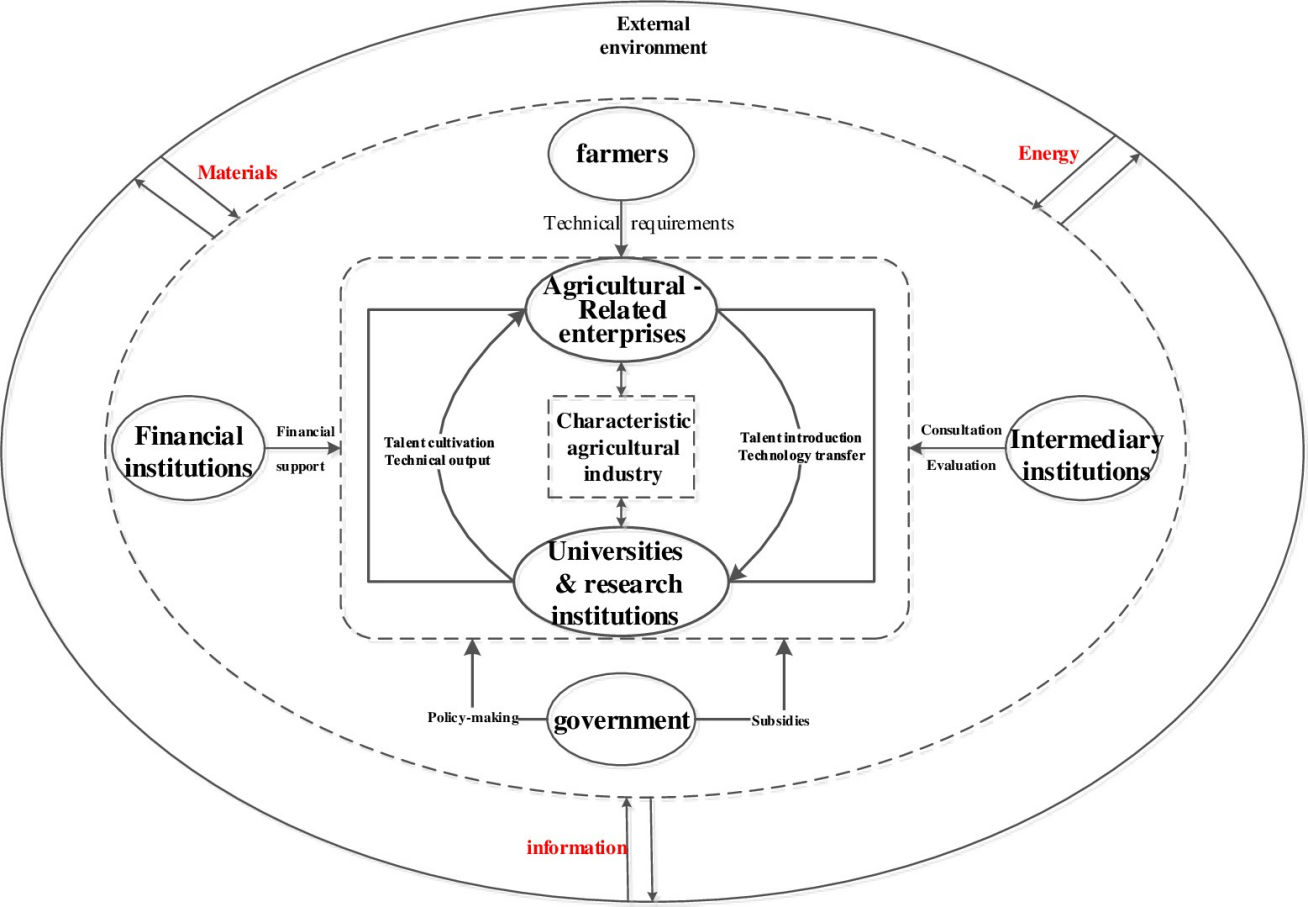
Integration framework for agricultural innovation ecosystem.

Agricultural innovation is a complex system involving the economy, society, and ecology. Addressing the problems of agriculture, rural areas, and farmers cannot be completed by a single subject, but must rely on the joint participation of multiple innovation subjects. The structural relationship between agricultural innovation ecosystem elements is depicted in Fig 1.

As seen from Fig 1, in the agricultural innovation ecosystem, industry-university-research is at the most central position. This is due to the limited independent innovation capacity of agriculture-related enterprises, which must rely on collaborative innovation among industry, academia and research to obtain sustainable innovation sources.

Agriculture-related enterprises constitute the basic unit of the agricultural industry, including not only those directly engaged in agricultural production, but also those providing various services for agricultural production and processing agricultural products [37]. As the main body of benefit innovation of the system, agriculture-related enterprises can create economic benefits for the system by absorbing agricultural intellectual property technologies and transforming them into innovative products [38]. The social benefits of the system may be improved through taxation and employment opportunities. In addition, agriculture-related enterprises can carry out agricultural production in a large-scale, intensive, and standardized way; make more effective use of agricultural resources; reduce agricultural non-point source pollution; and promote the improvement of system ecological benefits [39]. It can be seen that agriculture-related enterprises are the core group of an agricultural innovation ecosystem, who are the main actors maintaining the economic, social, and ecological benefits of the agricultural innovation ecosystem, as well as the dominant leaders and main driving force of agricultural innovation activities.

Universities & scientific research institutions are the providers of innovation achievements in an agricultural innovation ecosystem. Compared with other industries, agricultural R&D investment and the innovation talent reserve are significantly insufficient, while agriculture-related enterprises have limited independent innovation ability and, thus, lack the ability to fully undertake independent innovation [40]. They can only rely on the advantages of academic and research institutions, in terms of knowledge and technical personnel to support the agricultural innovation ecosystem to produce sustainable innovation. Colleges and universities are mainly engaged in the basic research of agriculture, which aims at innovating the frontiers of agricultural science theory. However, scientific research in colleges and universities should not be divorced from social needs, and should be guided by market demands. On the basis of industry–university–research cooperation, universities should possess a deep understanding of key technical problems, and then carry out basic research work in relevant areas [41]. In addition, agriculture-related colleges and universities shoulder the important task of training agricultural science and technology talents, thus providing a steady stream of high-quality talents for agricultural research and practice. Scientific research institutions are directly involved in the process of agricultural innovation. With the help of good platforms and resources provided by agriculture-related enterprises, scientific research institutions carry out the development of agricultural technology and the planning of research, transforming the achievements of agricultural scientific research into technological achievements that can be applied by enterprises and their industries, in order to enhance the content and added value of agricultural science and technology while promoting the high-quality development of agriculture.

The government acts as the strategy- and policy-maker and plays an important role, in terms of policy guidance and providing subsidies in the agricultural innovation ecosystem [42]. Due to the weak nature of agriculture itself, it is characterized by a long growth cycle, large investment, low industry barriers, and high risks. Besides the government, other innovation subjects are limited by objectives, interests, and resources, and may not be competent. Only the participation of the government can ensure the smooth development of innovation activities [33]. In the process of system operations, the government is not directly involved in agricultural technology innovation; however, it can, through system design and policy support, constrain and incentivize the innovation main body, fully combined with the local actual situation, in order to provide proactive strategies, clear agricultural innovation development directions, more targeted allocation of resources, and organization and coordination [43]. Therefore, in many cases, the government plays an important role in organizing and investing in agricultural innovation activities. When agricultural innovation resources are scarce, it is particularly important for the government to provide policy support, introduction of and investment into innovation resources, and social services.

In addition, financial institutions and intermediary institutions also play an indispensable role in the agricultural innovation ecosystem [44]. Financial institutions are the main support to promote agricultural innovation and provide indispensable financial support for the interior of the agricultural innovation ecosystem. Intermediary agencies play roles involving communication, coordination, integration, and promotion among multiple innovation subjects, and provide professional services such as agricultural technical consultation, evaluation, and industrialization of achievements for agriculture, rural areas, and farmers. Of course, farmers, as the main operating subject in agricultural development, are the demanders of agricultural innovation technology, and carry out re-innovation according to their actual agricultural technology demand, thus accelerating the sustainable development of the agricultural innovation ecosystem.

The innovation ecosystem is self-organizing and evolutionary [45], so is the agricultural innovation ecosystem as one of its branches. The innovation subjects and innovation elements in the agricultural innovation ecosystem interact with the external environment in the process of continuous development and change [46], which in turn causes structural changes in the whole system, leading to further renewal and enrichment of innovation subjects and innovation elements, expansion of the system scope and presentation of new environmental forms. The agricultural innovation ecosystem also has the attributes of a natural ecosystem. Through industry-university-research synergistic innovation, materials and energy in the natural ecosystem are transformed into economic materials and energy with exchange value, so as to promote the upgrading of science and technology [47]. Therefore, the agricultural innovation ecosystem will change and evolve dynamically over time, in a spiraling process from low level to high level. On this basis, it is of great significance to reveal the evolutionary relationship among innovation subjects in the agricultural innovation ecosystem from a dynamic perspective. However, existing studies mainly focus on static perspective analysis. In order to make up for the shortcomings of the existing research, and to deepen and expand the theoretical system of agricultural innovation ecosystem and evolution more systematically, the following is carried out in this paper: (1) We analyze the connotation of an agricultural innovation ecosystem, deconstruct its constituent elements and structure, and identify the role, relationship, and positioning of the collaborative innovation subject of the system; (2) The evolutionary game model is applied to explore and analyze the synergistic evolutionary behavior of the government, agriculture-related enterprises, universities & research institutions in order to explore the influencing factors of synergistic innovation [48]; and (3) the MATLAB software is used to conduct numerical simulation of the collaborative innovation evolution game of government, agriculture-related enterprises, universities & research institutions, in order to verify the effect of the collaborative innovation game process and influencing factors on collaborative innovation evolution of the agricultural innovation ecosystem. The relevant research results are expected to be helpful, in terms of reducing the opportunity cost and externalities of innovation subjects in the process of participating in synergistic innovation, improving the ability for collaborative innovation and co-evolution among subjects, guaranteeing the sustainable development of the system, and providing reference for the formulation of local government agricultural innovation development strategy planning.

## 3. Evolutionary game model of agricultural innovation ecosystem

### 3.1 Role identification

The development of regional agricultural innovation ecosystem in developed countries has been relatively mature, which provides valuable practical experience for our country. For example, the United States has built a sound innovation ecosystem with strong multinational companies such as Monsanto, Bunge and Novus International as the core, supported by universities, scientific research institutions and governments, and brought about the continuous emergence of modern agricultural products and technologies such as crop varieties, agricultural biotechnology, microbial technology and biological agents. It can be seen that the high degree of agricultural science and technology industrialization in developed countries is mainly due to the cultivation of a large number of enterprise innovation subjects integrating agricultural science and technology research and development and achievement transformation [49]. The practice of developed countries is more in line with the development process of the industrial innovation ecosystem in China, which relies mainly on large enterprises with strong strength as the core, complemented by innovative R&D in academic and research institutions. However, unlike developed countries, agricultural innovation ecosystem in China is in its infancy, and there is a lack of agricultural-related enterprises with independent innovation capabilities, which must rely on universities & research institutions as the main source of innovation to engage in industry-university-research cooperation. In addition, as a policy leader, although the government does not directly participate in the industry-university-research synergistic innovation, it will maintain the stability of the industry-university-research collaborative innovation through legal, economic and administrative means. Therefore, the roles of government, agriculture-related enterprises, universities & research institutions are indispensable in the agricultural innovation ecosystem.

In the agricultural innovation ecosystem, the collaborative innovation intention among the multiple subjects is usually long-term. Agriculture-related enterprises carry out in-depth cooperation with relevant innovation subjects and reach cooperation agreements. The collaborative innovation behaviors among agents is limited by these cooperation agreements, and is restricted and influenced by the strategy choices of all involved parties, each of which cannot fully realize the goal of maximizing their own interests. Thus, their collaborative innovation behaviors conform to the bounded rationality hypothesis of evolutionary game theory [50]. In this paper, the government, agriculture-related enterprises, universities, & research institutions are selected, in order to establish an evolutionary game model. The reason is that universities & research institutions have the talent, knowledge, and technology necessary for innovation, and can continuously provide new products, technologies, materials, and processes. Agriculture-related enterprises can provide material resources and locations for research and development, and can connect with the market, accurately grasp the market demand, in order to carry out effective and relevant industry–university–research innovation activities. The government can play a leading role in guiding industry, universities & research institutes to establish innovation cooperation based on market demand, so as to effectively promote agricultural innovation and development. All three can independently choose to participate or not to participate under the premise of limited rationality. Therefore, whether agriculture-related enterprises, universities & scientific research institutions choose collaborative innovation behaviors, and whether the government chooses to regulate collaborative innovation behaviors are in line with the dynamic process of evolutionary game, and are formed through multiple evolutionary games of various subjects. The reason why financial institutions and intermediaries are not selected is that they only provide funds, technology promotion and other services for agricultural innovation activities, play auxiliary service functions, and directly participate in the collaborative innovation of industry, university and research, and can not lead the success or failure of synergistic innovation. Therefore, this paper explores and analyzes the evolutionary game of collaborative innovation behaviors of government, agribusiness, universities and research institutions using the evolutionary game model in order to reveal the evolutionary process of agricultural innovation ecosystem.

### 3.2 Basic assumptions

This paper explores the evolution process of collaborative innovation behaviors of government, agriculture-related enterprises, universities & research institutions, and makes the following assumptions:

1. Game players
  Assuming that there are three game players, namely, the government, denoted as G, agriculture-related enterprises, denoted as A, and universities & research institutions, denoted as U. All three players carry out collaborative innovation on the basis of limited rationality, so they can not know the collaborative innovation behavior choices of the other two parties in advance. In order to explore the most favorable choice for themselves, they have to constantly adjust their collaborative innovation behaviors through practice [51].
2. Strategy selection.
  In the collaborative innovation strategy selection, each of the three game parties has two strategy choices [52]. On the one hand, the government can choose the strategy of regulation or non-regulation, i.e., the government’s game strategy set is (regulation, non-regulation). Regulation can be understood as the government supervising and regulating the collaborative innovation behaviors of agriculture-related enterprises, universities & research institutions through the formulation of policies and regulations, and providing subsidies and incentives to agriculture-related enterprises, universities & research institutions that actively participate in synergistic innovation. Assuming that the probability of the government adopting a strategy of regulation is *x*, the probability of adopting a strategy of no regulation is (1−*x*). On the other hand, agriculture-related enterprises, universities and research institutions can choose collaborative innovation or non-collaboration strategy according to their own interests, i.e. the set of game strategies of agriculture-related enterprises, universities & research institutions is (collaborative, non-collaborative). Assuming that the probability of agriculture-related enterprises, universities & research institutions participating in collaborative innovation is *y* and *z* respectively, the probability of not participating in collaborative innovation is (1−*y*) and (1−*z*) respectively. Among them, *x*,*y*,*z*∈[0,1]. In the game process, no matter the government or agriculture-related enterprises or universities & research institutions, any player is randomly paired to play the game.
3. Cooperation cost
  The government encourages agriculture-related enterprises, universities and research institutes to participate in collaborative innovation through policies, and regulates the process of synergistic innovation [53]. Assuming that the incentive cost generated is *K* and the cost of implementing regulation is *C*_*g*_. Among them, the proportion of government incentive funds received by agriculture-related enterprises participating in collaborative innovation is *γ*, and the proportion of government incentive funds received by universities & research institutes participating in collaborative innovation is (1−*γ*), *γ*∈[0,1]. When agricultural-related enterprises, universities & research institutions carry out synergistic innovation, both parties need to invest in human, material and financial resources. Assuming that the costs invested by agricultural-related enterprises and universities in adopting collaborative innovation strategies are *C*_*a*_ and *C*_*u*_ respectively. When the government imposes regulation and incentives on collaborative innovation activities, it will reduce the cost of collaborative innovation for agriculture-related enterprises, universities & research institutions. Assuming that when the government adopts the regulatory strategy, the cost reduction factor of collaborative innovation of agricultural-related enterprises is *ϕ*_1_, and the cost reduction factor of collaborative innovation of universities & research institutions is *ϕ*_2_. When the government adopts the regulatory strategy, the cost reduction of collaborative innovation of agriculture-related enterprises is *ϕ*_1_*C*_*a*_, and the cost reduction of collaborative innovation of universities and research institutions is *ϕ*_2_*C*_*u*_.
4. Cooperation benefits
  Government regulation and incentives can improve the efficiency of collaborative innovation between industry, academia and research, accelerate the speed of agricultural technology diffusion, and promote the transformation of regional agriculture to high-quality growth, allowing the government to reap the benefits. Assuming that *M*_*g*_ is the benefit obtained when the government adopts a regulatory strategy, and *N* is the benefit obtained when the government adopts a non-regulatory strategy. Agricultural-related enterprises, universities & scientific research institutions will generate basic income when they do not carry out synergistic innovation. Assuming that the base benefit of agriculture-related enterprises without collaborative innovation strategy is *I*_*a*_; the base benefit of universities & research institutions without collaborative innovation strategy is *I*_*u*_. In the case of synergistic innovation, both parties will gain additional collaborative innovation benefits. Assuming that the revenue generated by collaborative innovationof agriculture-related enterprises, universities and research institutions is *S*. Among them, the distribution ratio of collaborative innovation revenue of agriculture-related enterprises is *δ* respectively, and the distribution ratio of innovation revenue of universities and research institutions is (1−*δ*), *δ*∈[0,1]
5. Liquidated damages
  The prerequisite for collaborative innovation by agriculture-related enterprises, universities and research institutions is the signing of a contract and the payment of a deposit in advance.Under the guarantee of the contract, if any player betrays the contract involving agriculture-related enterprises, universities & scientific research institutions, it will pay the corresponding liquidated damages to the other player [54, 55]. Assuming that when the agriculture-related enterprises, universities & research institutions betray the synergistic innovation, the liquidated damages to be paid to the other player are *W*_*a*_ and *W*_*u*_ respectively.

### 3.3 Evolution model construction

Based on the above basic assumptions, the game payment matrix of the three parties in the agricultural innovation ecosystem when the government, agriculture-related enterprises, universities and research institutions adopt different strategies can be derived, as shown in Table 1.

**Table 1:** Payment matrix of three players in the collaborative innovation game.

Strategy Portfolio	Government	Agricultural-related enterprises	Universities & research institutions
(Regulation, Collaboration, Collaboration)	*M*_*g*_−*K*−*C*_*g*_	$I_{a}+\delta S+\gamma K-(1-\phi_{1})C_{a}$	$I_{u}+(1-\delta )S+(1-\gamma )K-(1-\phi_{2})C_{u}$
(Regulation, Non-collaboration, Collaboration)	*M*_*g*_−(1−*γ*)*K*−*C*_*g*_	*I*_*a*_−*W*_*a*_	$I_{u}+(1-\gamma )K+W_{a}-(1-\phi_{2})C_{u}$
(Regulation, Collaboration, Non-collaboration)	*M*_*g*_−*γK*−*C*_*g*_	$I_{a}+\gamma K+W_{u}-(1-\phi_{1})C_{a}$	*I*_*u*_−*W*_*u*_
(Regulation, Non-collaboration, Non-collaboration)	*N*−*C*_*g*_	*I*_*a*_	*I*_*u*_
(Non-regulation, Collaboration, Collaboration)	*N*	*I*_*a*_+*δS*−*C*_*a*_	*I*_*u*_+(1−*δ*)*S*−*C*_*u*_
(Non-regulation, Non-collaboration, Collaboration)	*N*	*I*_*a*_−*W*_*a*_	*I*_*u*_+*W*_*a*_−*C*_*u*_
(Non-regulation, Collaboration, Non-collaboration)	*N*	*I*_*a*_+*W*_*u*_−*C*_*a*_	*I*_*u*_−*W*_*u*_
(Non-regulation, Non-collaboration, Non-collaboration)	*N*	*I*_*a*_	*I*_*u*_

Based on the basic assumptions of the evolutionary game model and the payment matrix, an evolutionary game model of the agricultural innovation ecosystem can be constructed for the expected benefits of three parties: government (G), agriculture-related enterprises (A), universities & research institutions (U), as follows:

Assuming that the expected return when the government adopts a "regulation" strategy is *E*_*g*1_, the expected return when it adopts a "non-regulation" strategy is *E*_*g*2_, and the average expected return is ${\bar{E}}_{g}$, then:

$$\begin{array}{l} E_{g1}=yz(M_{g}-K-C_{g})+(1-y)z[M_{g}-(1-\gamma )K-C_{g}]\\ +y(1-z)(M_{g}-\gamma K-C_{g})+(1-y)(1-z)(M_{g}-C_{g}) \end{array}$$ (1)

$$E_{g2}=yzN+(1-y)zN+y(1-z)N+(1-y)(1-z)N$$ (2)

$${\bar{E}}_{g}=xE_{g1}+(1-x)E_{g2}$$ (3)

Assuming that the expected return of an agribusiness firm adopting a "collaboration" strategy is *E*_*a*1_, and the expected return of a "non-collaboration" strategy is *E*_*a*2_, the average expected return is ${\bar{E}}_{a}$, then:

$$\begin{array}{l} E_{a1}=xz[I_{a}+\delta S+\gamma K-(1-\phi_{1})C_{a}]+(1-x)z(I_{a}+\delta S-C_{a})\\ +x(1-z)[I_{a}+\gamma K+W_{u}-(1-\phi_{1})C_{a}]+(1-x)(1-z)(I_{a}+W_{u}-C_{a}) \end{array}$$ (4)

$$E_{a2}=xz(I_{a}-W_{a})+(1-x)z(I_{a}-W_{a})+x(1-z)I_{a}+(1-x)(1-z)I_{a}$$ (5)

$${\bar{E}}_{a}=yE_{a1}+(1-y)E_{a2}$$ (6)

Assuming that the expected return when universities & research institutions adopt the "Collaboration" strategy is *E*_*u*1_, and the expected return when adopting the "Non-collaboration" strategy is *E*_*u*2_, the average expected return is ${\bar{E}}_{u}$, then.


$$\begin{array}{l} E_{u1}=xy[I_{u}+(1-\delta )S+(1-\gamma )K-(1-\phi_{2})C_{u}]+(1-x)y[I_{u}+(1-\delta )S-C_{u}]\\ +x(1-y)[I_{u}+(1-\gamma )K+W_{a}-(1-\phi_{2})C_{u}]+(1-x)(1-y)(I_{u}+W_{a}-C_{u}) \end{array}$$ (7)

$$E_{u2}=xy(I_{u}-W_{u})+x(1-y)I_{u}+(1-x)y(I_{u}-W_{u})+(1-x)(1-y)I_{u}$$ (8)

$${\bar{E}}_{u}=zE_{u1}+(1-z)E_{u2}$$ (9)

From (1) and (3), the government’s replication dynamic equation can be constructed as follows:

$$\begin{array}{l} F(x)=x(E_{g1}-{\bar{E}}_{g})\\ =x(1-x)[M_{g}-C_{g}-N-y\gamma K-z(1-\gamma )K] \end{array}$$ (10)

Based on (4) and (6), the replication dynamic equation of agriculture-related enterprises can be constructed as follows:

$$\begin{array}{l} F(y)=\frac{dy}{dt}=y(E_{a1}-{\bar{E}}_{a})\\ =y(1-y)[x(\gamma K+\phi_{1}C_{a})+z(\delta S-W_{u}+W_{a})+W_{u}-C_{a}] \end{array}$$ (11)

From (7) and (9), the replication dynamics equations for universities and research institutions can be constructed as follows:

$$\begin{array}{l} F(z)=\frac{dz}{dt}=z(E_{u1}-{\bar{E}}_{u})\\ =z(1-z)\{x[(1-\gamma )K+\phi_{2}C_{u}]+y[(1-\delta )S+W_{u}-W_{a}]+W_{a}-C_{u}\} \end{array}$$ (12)

Simultaneous (10), (11) and (12), a replicated dynamic equation system for governments, agriculture-related enterprises, universities & research institutions can be established as follows:

$$\left\{ \begin{array}{l} F(x)=x(1-x)[M_{g}-C_{g}-N-y\gamma K-z(1-\gamma )K]\\ F(y)=y(1-y)[x(\gamma K+\phi_{1}C_{a})+z(\delta S-W_{u}+W_{a})+W_{u}-C_{a}]\\ f(z)=z(1-z)\{x[(1-\gamma )K+\phi_{2}C_{u}]+y[(1-\delta )S+W_{u}-W_{a}]+W_{a}-C_{u}\} \end{array}\right.$$ (13)

The three replication dynamic equations in system (13) reflect the direction and speed of strategic adjustment of government, agriculture-related enterprises, universities & scientific research institutions respectively [56].

### 3.4 Stability analysis of evolutionary game

According to the stability theory, when satisfying *F*(*x*) = 0, and $\frac{\partial F(x)}{\partial x}<0$; *F*(*y*) = 0, and $\frac{\partial F(y)}{\partial y}<0$; *F*(*z*) = 0, and $\frac{\partial F(z)}{\partial z}<0$, the government, agriculture-related enterprises, universities & research institutions strategy adjustment will be regionally stable.

(1) Stability analysis of government

When *F*(*x*) = 0, then there are two equilibrium points *x* = 0 or *x* = 1.


$$\frac{\partial F(x)}{\partial x}=(1-2x)[M_{g}-C_{g}-N-y\gamma K-z(1-\gamma )K]$$ (14)

**Fig 2:**
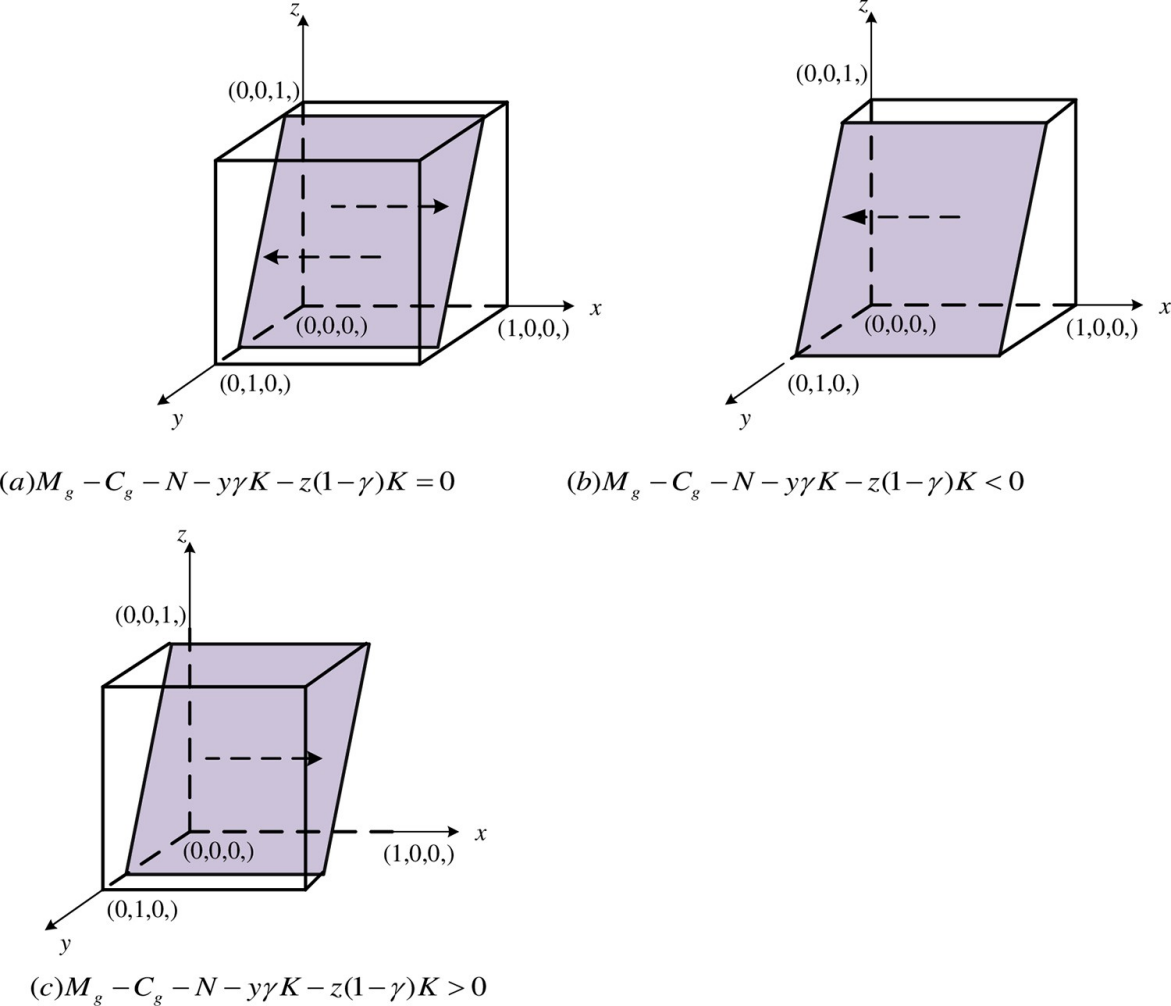
Dynamic phase diagram of the government. $(a)M_{g}-C_{g}-N-y\gamma K-z(1-\gamma )K=0$. $(b)M_{g}-C_{g}-N-y\gamma K-z(1-\gamma )K<0$. $(c)M_{g}-C_{g}-N-y\gamma K-z(1-\gamma )K>0$.

The strategic choices of the government during the evolutionary game of collaborative innovation in agricultural innovation ecosystems are as follows:

① When $M_{g}-C_{g}-N-y\gamma K-z(1-\gamma )K=0$, then $\frac{\partial F(x)}{\partial x}=0$ is constant, i.e., regardless of whether the government chooses to regulation or non-regulation, the benefits obtained by the same, *x* is in a steady state for any value within the interval of [0,1]. The replicated dynamic bitmap is plotted as shown in Fig 2(A).
② When $M_{g}-C_{g}-N-y\gamma K-z(1-\gamma )K<0$, then ${\frac{\partial F(x)}{\partial x}|}_{x=0}<0$, ${\frac{\partial F(x)}{\partial x}|}_{x=1}>0$. At this time, *x* = 0 is the final equilibrium point, i.e., when the net benefits obtained by the government by choosing the regulation strategy is greater than the benefits obtained by choosing non-regulation strategy. The government will eventually tend to choose regulation. The replication dynamic phase diagram is drawn as shown in the Fig 2(B).
③ When $M_{g}-C_{g}-N-y\gamma K-z(1-\gamma )K>0$, then ${\frac{\partial F(x)}{\partial x}|}_{x=0}>0$, ${\frac{\partial F(x)}{\partial x}|}_{x=1}<0$. At this time, *x* = 1 is the final equilibrium point, i.e., when the net benefits obtained by the government by choosing the regulation strategy is less than the benefits obtained by choosing non-regulation strategy. The government will eventually tend to choose regulation. The replication dynamic phase diagram is drawn as shown in the Fig 2(*C*).

(2) Stability analysis of agriculture-related enterprises

When *F*(*y*) = 0, then there are two equilibrium points *y* = 0 or *y* = 1.


$$\frac{\partial F(y)}{\partial y}=(1-2y)[x(\gamma K+\phi_{1}C_{a})+z(\delta S-W_{u}+W_{a})+W_{u}-C_{a}]$$ (15)

**Fig 3:**
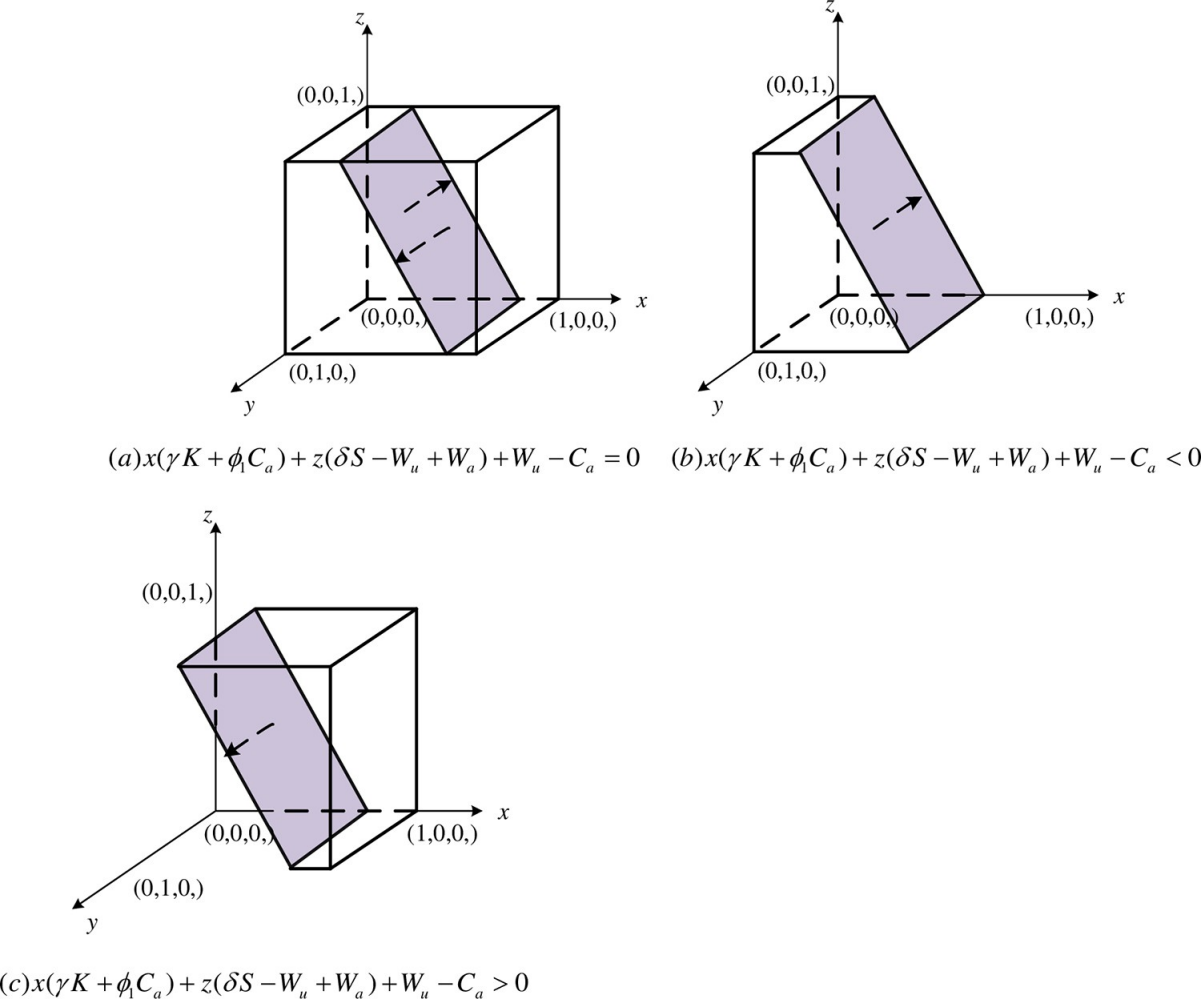
Dynamic phase diagram of agriculture-related enterprises. $(a)x(\gamma K+\phi_{1}C_{a})+z(\delta S-W_{u}+W_{a})+W_{u}-C_{a}=0$. $(b)x(\gamma K+\phi_{1}C_{a})+z(\delta S-W_{u}+W_{a})+W_{u}-C_{a}<0$. $(c)x(\gamma K+\phi_{1}C_{a})+z(\delta S-W_{u}+W_{a})+W_{u}-C_{a}>0$.

The strategic choices of agriculture-related enterprises during the evolutionary game of collaborative innovation in agricultural innovation ecosystems are as follows:

① When $x(\gamma K+\phi_{1}C_{a})+z(\delta S-W_{u}+W_{a})+W_{u}-C_{a}=0$, then $\frac{\partial F(y)}{\partial y}=0$ is constant, i.e, regardless of whether agriculture-related enterprises choose to collaboration or non-collaboration, *y* is in a stable state for any value in the interval of [0,1]. The replicated dynamic bitmap is plotted as shown in Fig 3(A).
② When $x(\gamma K+\phi_{1}C_{a})+z(\delta S-W_{u}+W_{a})+W_{u}-C_{a}<0$, ${\frac{\partial F(y)}{\partial y}|}_{y=0}<0$, ${\frac{\partial F(y)}{\partial y}|}_{y=1}>0$. At this time, *y* = 0 is the final equilibrium point, i.e., when the net benefits obtained by agriculture-related enterprises by choosing collaboration strategy is greater than the benefits obtained by choosing non-collaboration strategy. Agriculture-related enterprises will eventually tend to choose collaborative innovation and draw the replication dynamic phase diagram, as shown in Fig 3(B).
③ When $x(\gamma K+\phi_{1}C_{a})+z(\delta S-W_{u}+W_{a})+W_{u}-C_{a}>0$, ${\frac{\partial F(y)}{\partial y}|}_{y=0}>0$, ${\frac{\partial F(y)}{\partial y}|}_{y=1}<0$. At this time, *y* = 1 is the final equilibrium point, i.e., when the net benefits obtained by agriculture-related enterprises by choosing collaboration strategy is less than the benefits obtained by choosing non-collaboration strategy. Agriculture-related enterprises will eventually tend to choose not to participate in collaborative innovation, and the replication dynamic phase diagram will be drawn, as shown in Fig 3(*C*).

(3) Stability analysis of universities & research institutions

When *F*(*z*) = 0, then there are two equilibrium points *z* = 0 or *z* = 1.


$$\frac{\partial F(z)}{\partial z}=(1-2z)\{x[(1-\gamma )K+\phi_{2}C_{u}]+y[(1-\delta )S+W_{u}-W_{a}]+W_{a}-C_{u}\}$$ (16)

**Fig 4:**
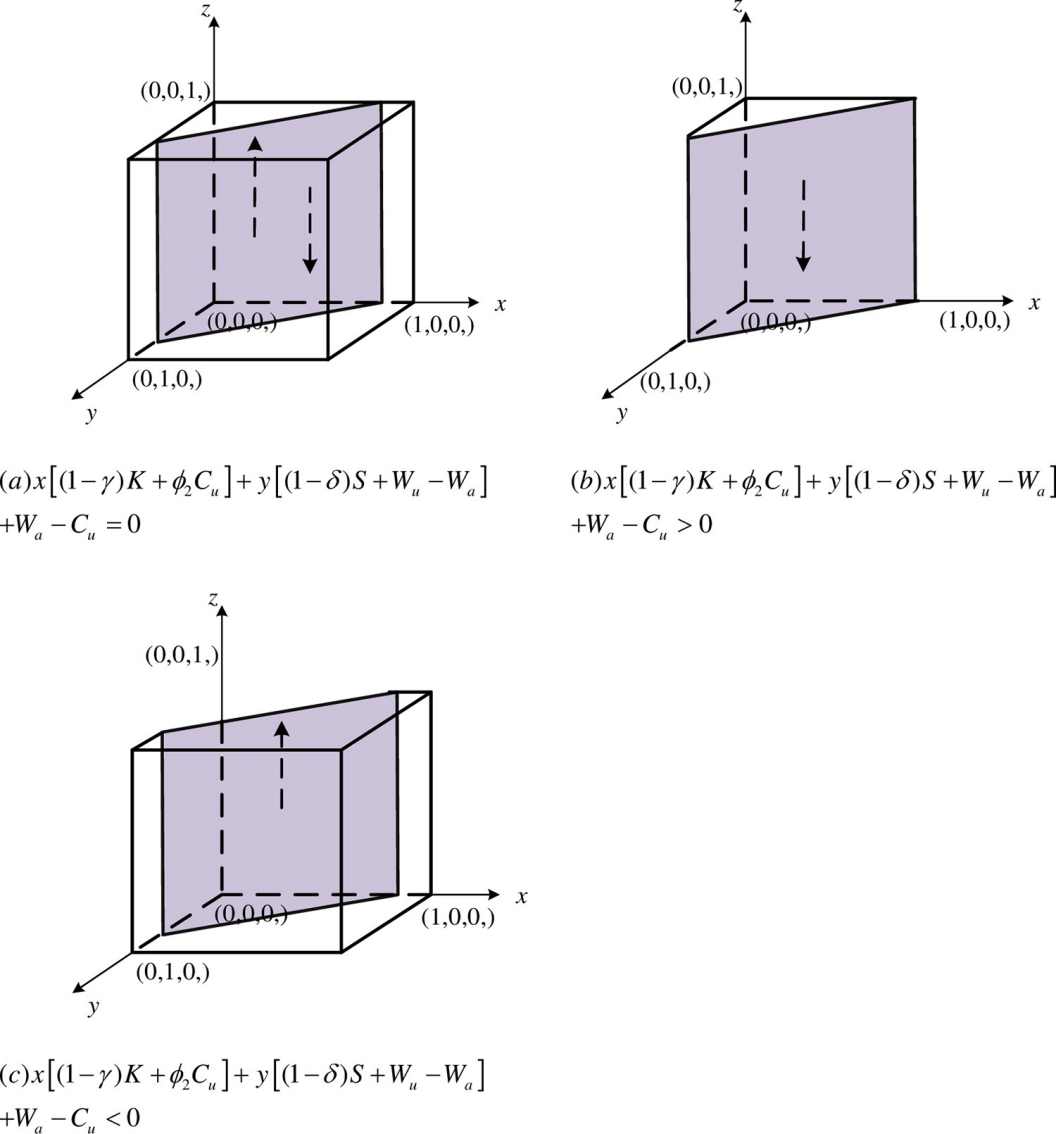
Dynamic phase diagram of universities & research institutions. $\begin{array}{l} \left(a)x[(1-\gamma )K+\phi_{2}C_{u}]+y[(1-\delta )S+W_{u}-W_{a}\right]\\ +W_{a}-C_{u}=0 \end{array}$. $\begin{array}{l} \left(b)x[(1-\gamma )K+\phi_{2}C_{u}]+y[(1-\delta )S+W_{u}-W_{a}\right]\\ +W_{a}-C_{u}>0 \end{array}$. $\begin{array}{l} \left(c)x[(1-\gamma )K+\phi_{2}C_{u}]+y[(1-\delta )S+W_{u}-W_{a}\right]\\ +W_{a}-C_{u}<0 \end{array}$.

The strategic choices of universities & research institutions during the evolutionary game of collaborative innovation in agricultural innovation ecosystems are as follows:

① When $x[(1-\gamma )K+\phi_{2}C_{u}]+y[(1-\delta )S+W_{u}-W_{a}]+W_{a}-C_{u}=0$, then $\frac{\partial F(z)}{\partial z}=0$ is constant, i.e, regardless of whether universities & research institutions choose to collaboration or non-collaboration, *z* is in a stable state for any value in the interval of [0,1]. The replicated dynamic bitmap is plotted as shown in Fig 4(A).
② When $x[(1-\gamma )K+\phi_{2}C_{u}]+y[(1-\delta )S+W_{u}-W_{a}]+W_{a}-C_{u}<0$, ${\frac{\partial F(z)}{\partial z}|}_{z=0}<0$, ${\frac{\partial F(z)}{\partial z}|}_{z=1}>0$. At this time, *z* =0 is the final equilibrium point, i.e., when the net benefits obtained by universities & research institutions by choosing collaboration strategy is greater than the benefits obtained by choosing non-collaboration strategy. Universities & research institutions will eventually tend to choose collaborative innovation and draw the replication dynamic phase diagram, as shown in Fig 4(B).
③ When $x[(1-\gamma )K+\phi_{2}C_{u}]+y[(1-\delta )S+W_{u}-W_{a}]+W_{a}-C_{u}>0$, ${\frac{\partial F(z)}{\partial z}|}_{z=0}>0$, ${\frac{\partial F(z)}{\partial z}|}_{z=1}<0$. At this time, *y* = 1 is the final equilibrium point, i.e., when the net benefits obtained by universities & research institutions by choosing collaboration strategy is less than the benefits obtained by choosing non-collaboration strategy. Universities & research institutions will eventually tend to choose not to participate in collaborative innovation, and the replication dynamic phase diagram will be drawn, as shown in Fig 4(C).

(4) Stability point analysis of tripartite evolutionary game

According to the research method of Friedman (1991), the stability analysis of the evolutionary equilibrium points of the three parties in the game can be carried out by establishing the Jacobi matrix of the replicated dynamic group [57].

Therefore, the Jacobi matrix of the above group of replicated dynamic system is as follows:

$$\left[\begin{array}{ccc} \begin{array}{c} (1-2x)[M_{g}-C_{g}-N\\ -y\gamma K-z(1-\gamma )K] \end{array} & x(1-x)\gamma K & -x(1-x)(1-\gamma )K\\ y(1-y)(\gamma K+\phi_{1}C_{a}) & \begin{array}{c} (1-2y)[x(\gamma K+\phi_{1}C_{a})\\ +z(\delta S-W_{u}+W_{a})+W_{u}-C_{a}] \end{array} & y(1-y)(\delta S-W_{u}+W_{a})+W_{u}-C_{a}\\ z(1-z)[(1-\gamma )K+\phi_{2}C_{u}] & z(1-z)[(1-\delta )S+W_{u}-W_{a}] & \begin{array}{c} (1-2z)\{x[(1-\gamma )K+\phi_{2}C_{u}]\\ +y[(1-\delta )S+W_{u}-W_{a}]+W_{a}-C_{u}\} \end{array} \end{array}\right]$$ (17)

In system (17), if *F*(*x*) = *F*(*y*) = *F*(*z*) = 0, 8 local equilibrium points of the collaborative innovation game can be obtained, which are: *O*_1_(0,0,0), *O*_2_(1,0,0), *O*_3_(0,1,0), *O*_4_(0,0,1), *O*_5_(1,1,0), *O*_6_(1,0,1), *O*_7_(0,1,1), *O*_8_(1,1,1). In an evolutionary game, the equilibrium point satisfying the Jacobi matrix when all eigenvalues are negative is the evolutionarily stable point of the game system [58]. Therefore, the corresponding Jacobi matrix of 8 equilibrium points are calculated respectively, and the eigenvalues of the matrix are obtained. In order to save space, only an example is shown.

When the equilibrium point is *O*_1_(0,0,0), the corresponding Jacobi matrix is as follows:

$$J_{1}=[\begin{array}{ccc} M_{g}-C_{g}-N & 0 & 0\\ 0 & W_{u}-C_{a} & 0\\ 0 & 0 & W_{a}-C_{u} \end{array}]$$ (18)

The eigenvalues of the Jacobi matrix can be obtained from (18) as *λ*_1_ = *M*_*g*_−*G*_*g*_−*N*, *λ*_2_ = *W*_*u*_−*C*_*a*_, *λ*_3_ = *W*_*a*_−*C*_*u*_. Similarly, Jacobi matrix eigenvalues corresponding to other equilibrium points can be calculated, as shown in Table 2.

**Table 2:** Eigenvalues of Jacobi matrix in evolutionary game.

Equilibrium points	*λ*_1_	*λ*_2_	*λ*_3_
*O*_1_(0,0,0)	*M*_*g*_−*C*_*g*_−*N*	*W*_*u*_−*C*_*a*_	*W*_*a*_−*C*_*u*_
*O*_2_(1,0,0)	−(*M*_*g*_−*C*_*g*_−*N*)	$\gamma K+W_{u}-(1-\phi_{1})C_{a}$	$(1-\gamma )K+W_{a}-(1-\phi_{2})C_{u}$
*O*_3_(0,1,0)	*M*_*g*_−*C*_*g*_−*N*−*γK*	*C*_*a*_−*W*_*u*_	$(1-\delta )S+W_{u}-C_{u}$
*O*_4_(0,0,1)	$\begin{array}{l} M_{g}-C_{g}-N\\ -(1-\gamma )K \end{array}$	*δS*+*W*_*a*_−*C*_*a*_	*C*_*u*_−*W*_*a*_
*O*_5_(1,1,0)	$\begin{array}{l} -(M_{g}-C_{g}-N\\ -\gamma K) \end{array}$	$-[\gamma K+W_{u}-(1-\phi_{1})C_{a}]$	$\begin{array}{l} (1-\gamma )K+(1-\delta )S\\ +W_{u}-(1-\phi_{2})C_{u} \end{array}$
*O*_6_(1,0,1)	$\begin{array}{l} -[M_{g}-C_{g}-N\\ -(1-\gamma )K] \end{array}$	*γK*+*δS*+*W* $\begin{array}{l} \gamma K+\delta S+W_{a}\\ -(1-\phi_{1})C_{a} \end{array}$	$\begin{array}{l} -[\left(1-\gamma \right)K+W_{a}\\ -(1-\phi_{a})C_{u}] \end{array}$
*O*_7_(0,1,1)	*M*_*g*_−*C*_*g*_−*N*−*K*	$-(\delta S+W_{a}-C_{a})$	$-[(1-\delta )S+W_{u}-C_{u}]$
*O*_8_(1,1,1)	−(*M*_*g*_−*C*_*g*_−*N*−*K*)	$\begin{array}{l} -[\gamma K+\delta S+W_{a}\\ -(1-\phi_{1})C_{a}] \end{array}$	$\begin{array}{l} -[\left(1-\gamma \right)K+(1-\delta )S\\ +W_{a}-(1-\phi_{2})C_{u}] \end{array}$

Through the analysis of Table 2, it is found that the positive and negative values of the three characteristic values corresponding to different strategy combinations are difficult to determine. This is because the evolutionary game process of the government, agriculture-related enterprises, universities & research institutions is affected by many factors, and the strategy selection of the three parties will be adjusted with the change of the influencing factors. In order to conform to the objective reality, suppose $M_{g}-C_{g}-N-K>0$, $\delta S+W_{a}-C_{a}>0$, $(1-\delta )S+W_{u}-C_{u}>0$, i.e., the net benefits obtained by the government by choosing regulation is greater than the benefits obtained by choosing non-regulation, and the net benefits obtained by agriculture-related enterprises, universities & research institutions by choosing collaboration is greater than the benefits obtained by choosing non-collaboration. In judging the positive and negative values of the 3 characteristic values corresponding to different strategy combinations, it is also necessary to consider the magnitude of the default amount obtained from the betrayal of co-innovation by only one player involved in co-innovation by the other player and the cost incurred by its participation in co-innovation. The specific situation is analyzed as follows:

(1) When *W*_*u*_>*C*_*a*_, *W*_*a*_>*C*_*u*_, it means that only one player of the agriculture-related enterprises or universities & research institutions involved in collaborative innovation obtains the default amount of the other player’s betrayal of collaborative innovation greater than the cost incurred by their participation in collaborative innovation, then the results of the stability analysis of the tripartite evolutionary game are shown in Table 3.

**Table 3:** Stability point analysis of tripartite evolutionary game for *W*_*u*_>*C*_*a*_, *W*_*a*_>*C*_*u*_.

Equilibrium points	*λ*_1_	*λ*_2_	*λ*_3_	Stability
*O*_1_(0,0,0)	+	+	+	Saddle point
*O*_2_(1,0,0)	-	+	+	Instability point
*O*_3_(0,1,0)	+	-	+	Instability point
*O*_4_(0,0,1)	+	+	-	Instability point
*O*_5_(1,1,0)	-	-	+	Instability point
*O*_6_(1,0,1)	-	+	-	Instability point
*O*_7_(0,1,1)	+	-	-	Instability point
*O*_8_(1,1,1)	-	-	-	*ESS*

According to the analysis results in Table 3, it can be seen that when *W*_*u*_>*C*_*a*_, *W*_*a*_>*C*_*u*_, in the evolutionary game of agricultural innovation ecosystem, point *O*_1_(0,0,0) is the saddle point, the stable behavior combination of the evolutionary game occurs at point *O*_8_(1,1,1), and the rest are instability points. It shows that when *W*_*u*_>*C*_*a*_, *W*_*a*_>*C*_*u*_, the result of the long-term game among the three players is that the government will choose to impose regulation on collaborative innovation, and the agriculture-related enterprises, universities & research institutions will choose collaborative innovation, i.e., the strategy combination of the equilibrium point is (regulation, collaboration, collaboration).

(2) When *W*_*u*_>*C*_*a*_, *W*_*a*_>*C*_*u*_, it means that the cost generated by the participation of agriculture-related enterprises in collaborative innovation is smaller than the default payment made by universities & research institutions betraying collaborative innovation, meanwhile, the cost generated by the participation of universities & research institutions in collaborative innovation is smaller than the default payment made by agriculture-related enterprises betraying collaborative innovation. The discussion can be divided into two cases., and the stability analysis results of the tripartite evolutionary game are shown in Table 4.

**Table 4:** Stability point analysis of tripartite evolutionary game for *W*_*u*_>*C*_*a*_, *W*_*a*_<*C*_*u*_.

Equilibrium points	$(1-\gamma )K+W_{a}-(1-\phi_{2})C_{u}>0$				$(1-\gamma )K+W_{a}-(1-\phi_{2})C_{u}<0$			
	*λ*_1_	*λ*_2_	*λ*_3_	Stability	*λ*_1_	*λ*_2_	*λ*_3_	Stability
*O*_1_(0,0,0)	+	+	-	Instability point	+	+	-	Instability point
*O*_2_(1,0,0)	-	+	+	Instability point	-	+	-	Instability point
*O*_3_(0,1,0)	+	-	+	Instability point	+	-	+	Instability point
*O*_4_(0,0,1)	+	+	+	Saddle point	+	+	+	Saddle point
*O*_5_(1,1,0)	-	-	+	Instability point	-	-	+	Instability point
*O*_6_(1,0,1)	-	+	-	Instability point	-	+	+	Instability point
*O*_7_(0,1,1)	+	-	-	Instability point	+	-	-	Instability point
*O*_8_(1,1,1)	-	-	-	*ESS*	-	-	-	*ESS*

According to the analysis results in Table 4, when *W*_*u*_>*C*_*a*_, *W*_*a*_>*C*_*u*_, two cases $(1-\gamma )K+W_{a}-(1-\phi_{2})C_{u}>0$ and $(1-\gamma )K+W_{a}-(1-\phi_{2})C_{u}<0$ should be considered to judge the positive and negative eigenvalues of different strategy combinations, i.e., the sum of the government incentive funds obtained and the liquidated damages paid by the other player and the cost of collaborative innovation under government regulation should be compared when universities & research institutions choose collaboration strategy separately. The analysis shows that although the positive and negative eigenvalues of different equilibrium points are different in both cases, the saddle point is *O*_4_(0,0,1), the final stable point is *O*_8_(1,1,1), and the rest are instability points. When *W*_*u*_>*C*_*a*_, *W*_*a*_>*C*_*u*_, the stable strategy combination of the tripartite long-term game is (regulation, collaboration, collaboration).

(3) When *W*_*u*_<*C*_*a*_, *W*_*a*_>*C*_*u*_, it means that the cost generated by the participation of agriculture-related enterprises in collaborative innovation is greater than the default paid by universities & research institutions betraying collaborative innovation, meanwhile, the cost generated by the participation of universities & research institutions in collaborative innovation is smaller than the default paid by agriculture-related enterprises betraying collaborative innovation, and the same can be discussed in two cases, and the stability analysis results of the tripartite evolutionary game are shown in Table 5.

**Table 5:** Stability point analysis of tripartite evolutionary game for *W*_*u*_<*C*_*a*_, *W*_*a*_>*C*_*u*_.

Equilibrium points	$\gamma K+W_{u}-(1-\phi_{1})C_{a}>0$				$\gamma K+W_{u}-(1-\phi_{1})C_{a}<0$			
	*λ*_1_	*λ*_2_	*λ*_3_	Stability	*λ*_1_	*λ*_2_	*λ*_3_	Stability
*O*_1_(0,0,0)	+	-	+	Instability point	+	-	+	Instability point
*O*_2_(1,0,0)	-	+	+	Instability point	-	-	+	Instability point
*O*_3_(0,1,0)	+	+	+	Saddle point	+	+	+	Saddle point
*O*_4_(0,0,1)	+	+	-	Instability point	+	+	-	Instability point
*O*_5_(1,1,0)	-	-	+	Instability point	-	-	+	Instability point
*O*_6_(1,0,1)	-	+	-	Instability point	-	+	-	Instability point
*O*_7_(0,1,1)	+	-	-	Instability point	+	-	-	Instability point
*O*_8_(1,1,1)	-	-	-	*ESS*	-	-	-	*ESS*

According to the analysis results in Table 5, when *W*_*u*_<*C*_*a*_, *W*_*a*_>*C*_*u*_, two cases $\gamma K+W_{u}-(1-\phi_{1})C_{a}>0$ and $\gamma K+W_{u}-(1-\phi_{1})C_{a}<0$ should be considered to judge the positive and negative eigenvalues of different strategy combinations, i.e., the sum of the government incentive funds obtained and the liquidated damages paid by the other player and the cost of collaborative innovation under government regulation when the agriculture-related enterprises choose the collaboration strategy separately. The analysis shows that although the positive and negative eigenvalues of different equilibrium points are different in both cases, the saddle point is *O*_3_(0,1,0), the final stable point is *O*_8_(1,1,1), and the rest are unstable points. When *W*_*u*_<*C*_*a*_, *W*_*a*_>*C*_*u*_, the stable strategy combination of the tripartite long-term game is (regulation, collaboration, collaboration).

(4) When *W*_*u*_<*C*_*a*_, *W*_*a*_<*C*_*u*_, it means that the cost of agriculture-related enterprises participating in collaborative innovation is greater than the liquidated damages paid by universities & research institutions for betraying collaborative innovation, meanwhile, the cost of universities & research institutions participating in collaborative innovation is also greater than the liquidated damages paid by agriculture-related enterprises for betraying collaborative innovation, which needs to be discussed in four cases. stability analysis results of the tripartite evolutionary game are shown in Table 6.

**Table 6:** Stability point analysis of tripartite evolutionary game for *W*_*u*_<*C*_*a*_, *W*_*a*_<*C*_*u*_.

Equilibrium points	$\gamma K+W_{u}-(1-\phi_{1})C_{a}>0$ $(1-\gamma )K+W_{a}-(1-\phi_{2})C_{u}>0$				$\gamma K+W_{u}-(1-\phi_{1})C_{a}>0$ $(1-\gamma )K+W_{a}-(1-\phi_{2})C_{u}<0$			
	*λ*_1_	*λ*_2_	*λ*_3_	Stability	*λ*_1_	*λ*_2_	*λ*_3_	Stability
*O*_1_(0,0,0)	+	-	-	Instability point	+	-	-	Instability point
*O*_2_(1,0,0)	-	+	+	Instability point	-	+	-	Instability point
*O*_3_(0,1,0)	+	+	+	Saddle point	+	+	+	Saddle point
*O*_4_(0,0,1)	+	+	+	Saddle point	+	+	+	Saddle point
*O*_5_(1,1,0)	-	-	+	Instability point	-	-	+	Instability point
*O*_6_(1,0,1)	-	+	-	Instability point	-	+	+	Instability point
*O*_7_(0,1,1)	+	-	-	Instability point	+	-	-	Instability point
*O*_8_(1,1,1)	-	-	-	*ESS*	-	-	-	*ESS*
Equilibrium points	$\gamma K+W_{u}-(1-\phi_{1})C_{a}<0$ $(1-\gamma )K+W_{a}-(1-\phi_{2})C_{u}>0$				$\gamma K+W_{u}-(1-\phi_{1})C_{a}<0$ $(1-\gamma )K+W_{a}-(1-\phi_{2})C_{u}<0$			
	*λ*_1_	*λ*_2_	*λ*_3_	Stability	*λ*_1_	*λ*_2_	*λ*_3_	Stability
*O*_1_(0,0,0)	+	-	-	Instability point	+	-	-	Instability point
*O*_2_(1,0,0)	-	-	+	Instability point	-	-	-	*ESS*
*O*_3_(0,1,0)	+	+	+	Saddle point	+	+	+	Saddle point
*O*_4_(0,0,1)	+	+	+	Saddle point	+	+	+	Saddle point
*O*_5_(1,1,0)	-	+	+	Instability point	-	-	+	Instability point
*O*_6_(1,0,1)	-	+	-	Instability point	-	+	-	Instability point
*O*_7_(0,1,1)	+	-	-	Instability point	+	-	-	Instability point
*O*_8_(1,1,1)	-	-	-	*ESS*	-	-	-	*ESS*

According to the analysis results in Table 6, when *W*_*u*_<*C*_*a*_, *W*_*a*_<*C*_*u*_, in order to judge the merits of different strategy combinations, it is necessary to further compare the sum of the government incentive funds received and the liquidated damages paid by the other player and the cost of collaborative innovation under government regulation when one of the agriculture-related enterprises or universities & research institutions chooses collaboration strategy separately. Therefore, it can be divided into four cases: $\gamma K+W_{u}-(1-\phi_{1})C_{a}>0$, $(1-\gamma )K+W_{a}-(1-\phi_{2})C_{u}>0$; $\gamma K+W_{u}-(1-\phi_{1})C_{a}>0$, $(1-\gamma )K+W_{a}-(1-\phi_{2})C_{u}<0$; $\gamma K+W_{u}-(1-\phi_{1})C_{a}<0$, $(1-\gamma )K+W_{a}-(1-\phi_{2})C_{u}>0$; $\gamma K+W_{u}-(1-\phi_{1})C_{a}<0$, $(1-\gamma )K+W_{a}-(1-\phi_{2})C_{u}<0$. The analysis shows that saddle points in all four cases were *O*_3_(0,1,0) and *O*_4_(0,0,1). Except for $\gamma K+W_{u}-(1-\phi_{1})C_{a}<0$, $(1-\gamma )K+W_{a}-(1-\phi_{2})C_{u}<0$, the stable point of the equilibrium point in the other three cases is *O*_8_(1,1,1), i.e., the stable strategy combination of the tripartite long-term game is (regulation, collaboration, collaboration). In the case of $\gamma K+W_{u}-(1-\phi_{1})C_{a}<0$, $(1-\gamma )K+W_{a}-(1-\phi_{2})C_{u}<0$, the stable points of the equilibrium point are *O*_2_(1,0,0) and *O*_8_(1,1,1), i.e., the stable strategy combination of the tripartite long-term game is (regulation, non-collaboration, non-collaboration) or (regulation, collaboration, collaboration). In short, when *W*_*u*_<*C*_*a*_, *W*_*a*_<*C*_*u*_, as long as the sum of government incentive funds obtained by at least one of the agriculture-related enterprises or universities & research institutions for selecting synergy strategies separately and the liquidated damages paid by the other player is greater than the cost of collaborative innovation generated under government regulation, the three parties of the government, agriculture-related enterprises, universities & research institutions will choose (regulation, collaboration, collaboration); When the sum of government incentive funds obtained by agriculture-related enterprises or universities & research institutions and the liquidated damages paid by the other player are both less than the cost of collaborative innovation generated under government regulation, the three players of the government, agriculture-related enterprises, universities & research institutions may choose (regulation, non-collaboration, non-collaboration) or (regulation, collaboration, collaboration). The final result of the game depends on the reward and punishment mechanism among agriculture-related enterprises, universities & research institutions and the government’s regulation.

To sum up, this paper attempts to explore the three parties’ evolutionary game strategy choices of government, agriculture-related enterprises, universities & research institutions under different conditions. It is found that under the premise that the net benefits of the government regulation are greater than those of non-regulation. At the same time, on the premise that the net benefits of agriculture-related enterprises, universities & research institutions choosing collaboration are greater than the benefits of choosing non-collaboration. As long as the sum of at least one player of agriculture-related enterprises or universities & research institutions choosing collaboration strategy independently to obtain government incentive funds and the other player paying liquidated damages is greater than the cost of collaborative innovation under government regulation, all three players will ultimately choose (regulation, collaboration, collaboration). Only when the sum of the government incentive funds and the liquidated damages paid by the other player is less than the cost of collaborative innovation under government regulation, the three players may choose (regulation, non-collaboration, non-collaboration) or (regulation, collaboration, collaboration). The game players of the agricultural innovation ecosystem all pursue the maximization of benefits, i.e., (regulation, collaboration, collaboration) is a combination of strategies beneficial to the government, agriculture-related enterprises, universities & research institutions. Through collaborative innovation, agriculture-related enterprises can transform new technologies into new products and improve social and economic benefits. Universities & research institutions can better realize technology transformation. At the same time, the social benefits generated by collaborative innovation can enhance the recognition of the government in the minds of the public, thus prompting the government to adopt active policies to support collaborative innovation activities. Therefore, the optimal combination of (regulation, collaboration, collaboration) strategy, i.e., the equilibrium point *O*_8_(1,1,1) is the optimal state. In order to promote industry-university-research collaborative innovation, agriculture-related enterprises, universities & research institutions should establish a more perfect reward and punishment mechanism, reasonably control costs, and establish a perfect liquidated damages system for breach of contract. The government should strengthen regulation and actively guide the atmosphere of collaborative innovation.

## 4. Evolutionary game numerical simulation analysis

In recent years, China’s agricultural supply-side structural reform had continued to advance, and the quality, efficiency and competitiveness of agriculture have been continuously improved. According to the data of “China Science and Technology Statistical Yearbook 2022”, in 2021, there were 993 agricultural science research and development institutions in China, with 67,881 R&D personnel and internal R&D expenditure of 250,74.25 million yuan. 34,602 agricultural science and technology papers had been published, 12,189 patents had been applied for, and 45,548 science and technology market contracts had been concluded. Total output value of agriculture, forestry, animal husbandry and fisheries reached 14.7013 trillion yuan. These achievements were attributed to the gradual improvement of the agricultural innovation ecosystem and the continuous deepening of industry-university-research collaborative innovation. According to the actual situation of collaborative innovation, the following initial values are assigned to each parameter variable, and the unit is unified into millions of yuan: *M*_*g*_ = 18, *K* =5, *C*_*g*_ = 6, *I*_*a*_ = 15, *I*_*u*_ = 10, *S* = 8, *C*_*a*_ = 10, *C*_*u*_ = 8, *δ* = 0.5, *γ* = 0.5, *ϕ*_1_ = 0.2, *ϕ*_2_ = 0.3, *W*_*a*_ = 3, *W*_*u*_ = 2, *N* =5. In this paper, MATLA2016a is used for simulation analysis.

(1) The influence of simultaneous changes in the willingness of the government, agriculture-related enterprises, universities & research institutions

**Fig 5:**
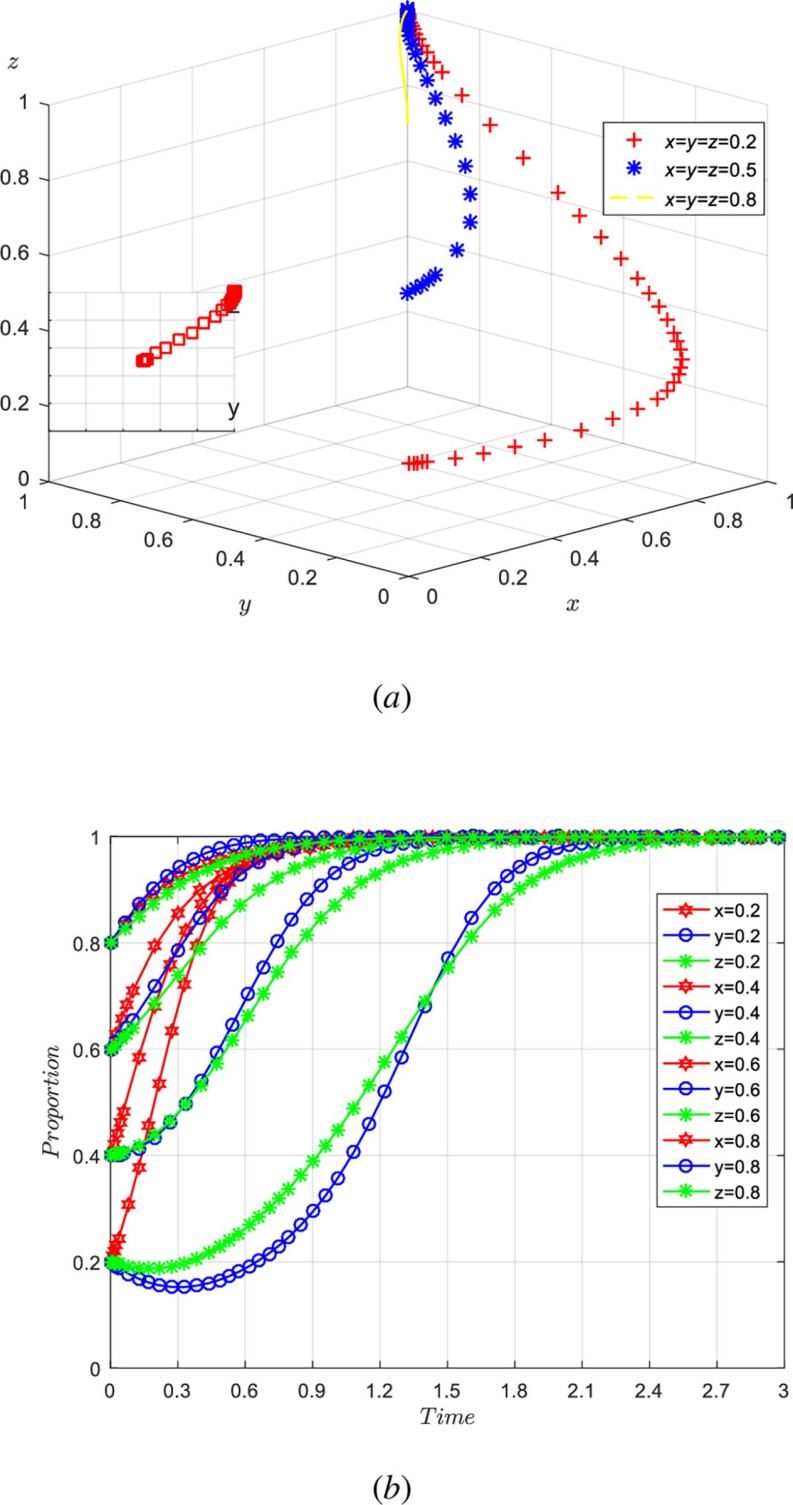
Influence of simultaneous changes of x, y, z on evolution results.

Assuming *x* =*y* = *z* = 0.2, *x* =*y* = *z* = 0.4, *x* =*y* = *z* = 0.6, *x* =*y* = *z* = 0.8, i.e., when the participation intentions of the government, agriculture-related enterprises, universities & research institutions change at the same time, the evolution results of the equilibrium strategy are shown in Fig 5.

As shown in Fig 5, the initial values are constant, and the willingness of the government, agriculture-related enterprises, universities & research institutions converges to 1 at different levels with different speeds. When the tripartite willingness is between 0.2 and 0.4, the speed at which the government’s willingness to implement regulation converges to 1 is higher than the cooperative willingness of agriculture-related enterprises, universities & research institutions. With the gradual increase of the tripartite willingness, the speed at which the agriculture-related enterprises, colleges and research institutions converges to 1 is accelerated, while the speed at which the government converges to 1 is slowing down. When the tripartite willingness exceeds 0.6, the willingness of agriculture-related enterprises to participate in collaborative innovation catches up with and exceeds the willingness of the government. When the tripartite willingness exceeds 0.8, the willingness of universities & research institutions to participate in collaborative innovation also catches up with and exceeds the willingness of the government. The simulation results show that when the tripartite willingness is not high, it is necessary for the government to play a more active role to encourage agriculture-related enterprises, universities & research institutions to participate in collaborative innovation through policy guidance, so as to promote the benign development of agricultural innovation ecosystem. As the beneficiary groups of agricultural innovation ecosystem gradually increase, the willingness of agriculture-related enterprises, universities & research institutions to collaborate on innovation will also increase, and the dividends brought by collaborative innovation will exceed government incentives. At this time, the market mechanism plays a major role, while the role of the government is weakening, and the government only needs to properly regulate and promote the collaborative cooperation of agriculture-related enterprises, universities & research institutions.

(2) The influence of individual changes in the willingness of the government, agriculture-related enterprises, universities & research institutions

**Fig 6:**
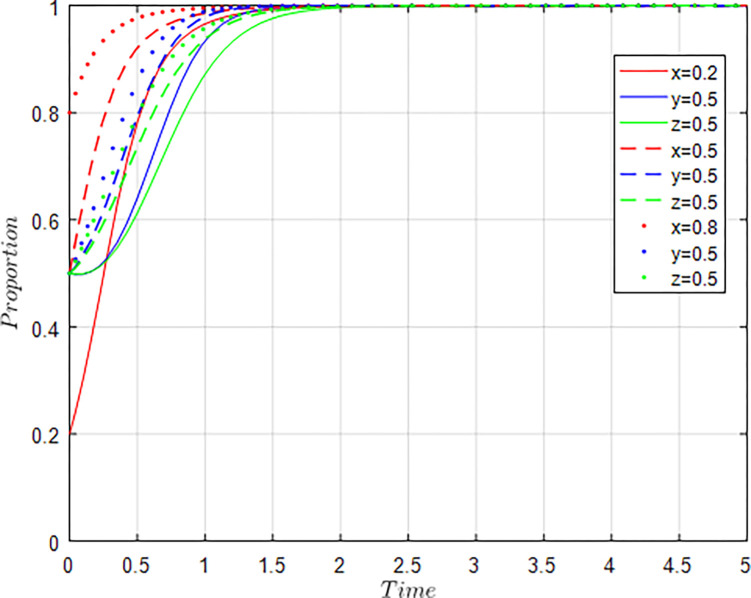
Influence of x changes on evolution results.

Assuming *x* = 0.2, *y* = 0.5, *z* = 0.5; *x* = 0.5, *y* = 0.5, *z* = 0.5; *x* = 0.8, *y* = 0.5, *z* = 0.5, i.e, the willingness of agriculture-related enterprises, universities & research institutions remains at the level of 0.5, and the willingness of the government is constantly changing. The evolution results of the equilibrium strategy are shown in Fig 6.

As shown in Fig 6, when the government’s regulatory willingness is lower, the slower the willingness of agriculture-related enterprises, universities & research institutions to participate in collaborative innovation converges to 1. With the increase of government regulatory willingness, the speed of the willingness of agriculture-related enterprises, universities & research institutions to participate in collaborative innovation converges to 1 is accelerated, and the willingness of agriculture-related enterprises to participate in collaborative innovation is always higher than that of universities & research institutions. When the government’s regulatory willingness exceeds 0.8, the convergence rate of agriculture-related enterprises, universities & research institutions to 1 is faster than that of the government. The simulation results show that it is very necessary for the government to strengthen regulation to promote the collaborative innovation evolution of the agricultural innovation ecosystem, and the government to increase regulation cost and incentive cost can effectively promote the collaborative innovation of agriculture-related enterprises, universities & research institutions, especially for agriculture-related enterprises. However, with the increase of the government’s willingness to regulate, the role of the government is weakening, which is consistent with the argument above.

**Fig 7:**
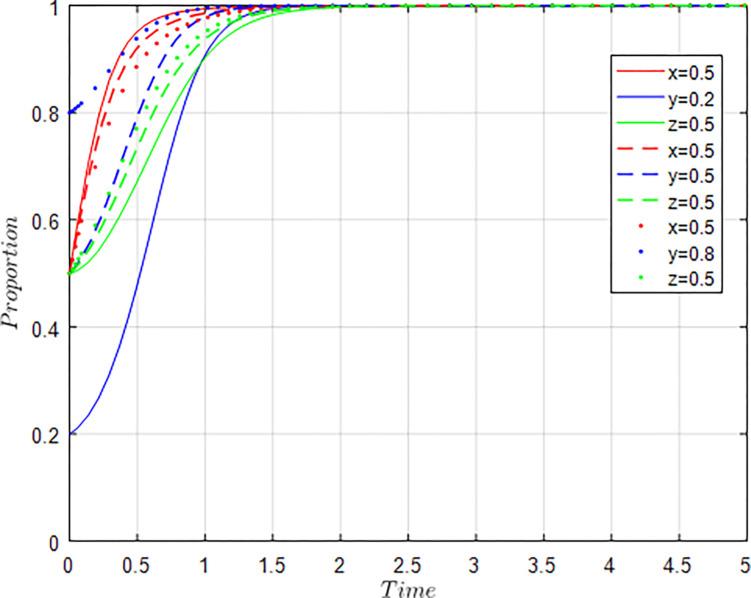
Influence of y changes on evolution results.

Assuming *x* = 0.5, *y* = 0.2, *z* = 0.5; *x* = 0.5, *y* = 0.5, *z* = 0.5; *x* = 0.5, *y* = 0.8, *z* = 0.5, i.e, The willingness of the government, universities & research institutions remains at the level of 0.5, while the willingness of agriculture-related enterprises is constantly changing. The evolution results of equilibrium strategy are shown in Fig 7.

As shown in Fig 7, the higher the willingness of agriculture-related enterprises to participate in collaborative innovation, the faster the willingness of universities & research institutions to participate in collaborative innovation converges to 1. However, with the increase of the participation willingness of agriculture-related enterprises, the speed of the government’s regulatory willingness to converge to 1 is constantly declining, and the speed of the government’s decline is less than the speed of the increase of agriculture-related enterprises, universities & research institutions. The simulation results show that as the willingness of agriculture-related enterprises to participate in collaborative innovation increases, agriculture-related enterprises will actively seek cooperation with universities & research institutions, so as to promote the improvement of the willingness of universities & research institutions to participate in collaborative innovation. In the process of collaborative evolution, the government needs to play a policy regulatory role. However, when the collaborative evolution of agricultural innovation ecosystem enters a virtuous cycle, the role of the government will continue to weaken.

**Fig 8:**
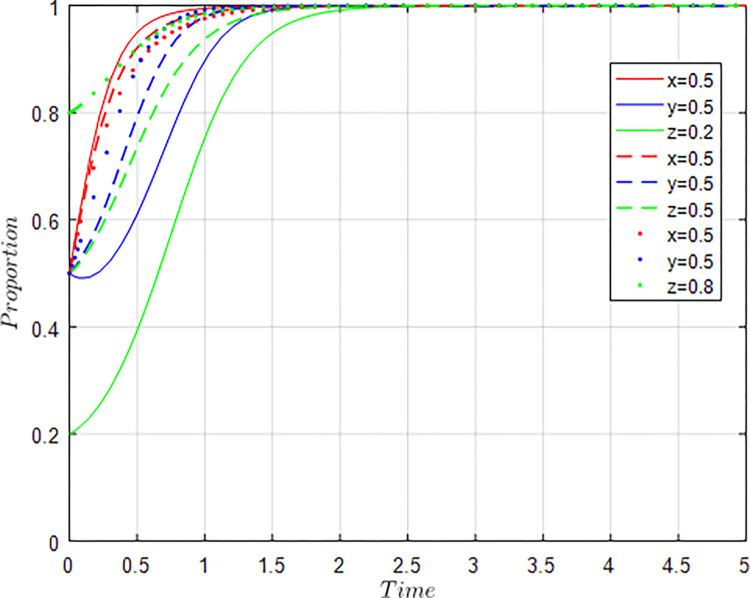
Influence of z changes on evolution results.

Assuming *x* = 0.5, *y* = 0.5, *z* = 0.2; *x* = 0.5, *y* = 0.5, *z* = 0.5; *x* = 0.5, *y* = 0.5, *z* = 0.8, i.e, the willingness of the government and agriculture-related enterprises remains at 0.5, and the willingness of universities & research institutions is constantly changing, the evolution results of the equilibrium strategy are shown in Fig 8.

As shown in Fig 8, the higher the willingness of universities & research institutions to participate in collaborative innovation, the faster the willingness of agriculture-related enterprises to participate in collaborative innovation converges to 1, and the speed of the willingness of agriculture-related enterprises to converge to 1 is always higher than the speed of the willingness of universities and research institutions to converge to 1. Similar to the change in the unilateral willingness of agriculture-related enterprises, when the participation willingness of universities & research institutions increases, the speed at which the government’s regulatory willingness converges to 1 is also declining. The simulation results show that as the providers of innovation achievements of universities & research institutions increase their willingness to participate in collaborative innovation, the benefits of agriculture-related enterprises from industry-university-research collaborative innovation will increase significantly, thus promoting the participation of agriculture-related enterprises in collaborative innovation. As the direct beneficiaries of innovation results, agriculture-related enterprises are significantly more willing to participate. When the collaborative innovation relationship tends to be stable, the role of the government will continue to weaken, and the market mechanism will play a more active role.

(3) The influence of changes in government regulatory policies

The role of the government in the collaborative evolution of the agricultural innovation ecosystem includes supervising and regulating the collaborative innovation behavior of agriculture-related enterprises, universities & research institutions, and providing subsidies and incentives to those enterprises, universities & research institutions that actively participate in collaborative innovation.

**Fig 9:**
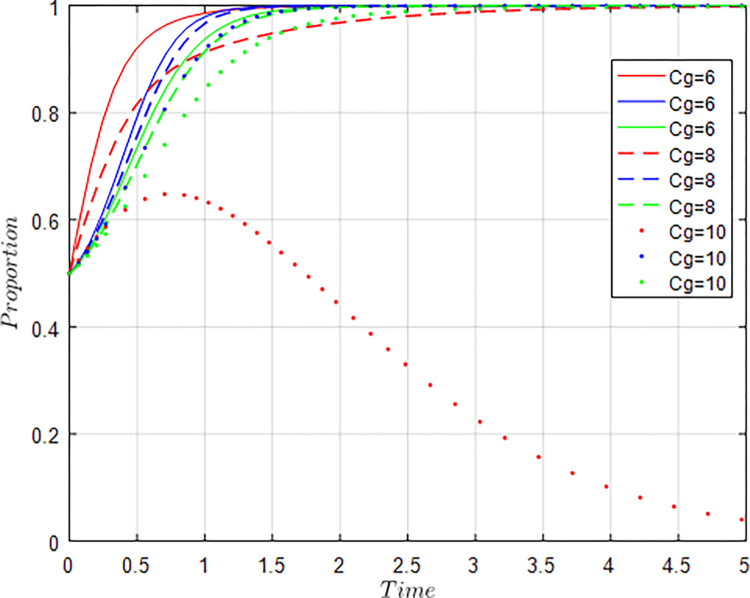
Influence of *C*_*g*_ changes on evolution results.

All other things being equal, let *C*_*g*_ = 6; *C*_*g*_ = 8; *C*_*g*_ = 10 respectively, i.e., other conditions remain unchanged, the cost of government regulation changes, and the evolution result of equilibrium strategy is shown in Fig 9.

As shown in Fig 9, the threshold of government regulation cost is between 8 and 10. When the government regulation cost is greater than this threshold, the government’s willingness to implement regulation will converge to 0, i.e., the government will choose the non-regulation strategy. Instead, the government will choose a regulatory strategy. Meanwhile, with the increase of government regulatory costs, the willingness of agriculture-related enterprises, universities & research institutions to participate in collaborative innovation converges to 1. The simulation results show that the increase of government regulatory costs reduces the government’s willingness to implement regulation, and also indirectly affects the enthusiasm of agriculture-related enterprises, universities & research institutions to participate in collaborative innovation. Excessive regulatory costs will bring unnecessary human and material costs to the government, thus forcing the government to adopt non-regulation strategy.

**Fig 10:**
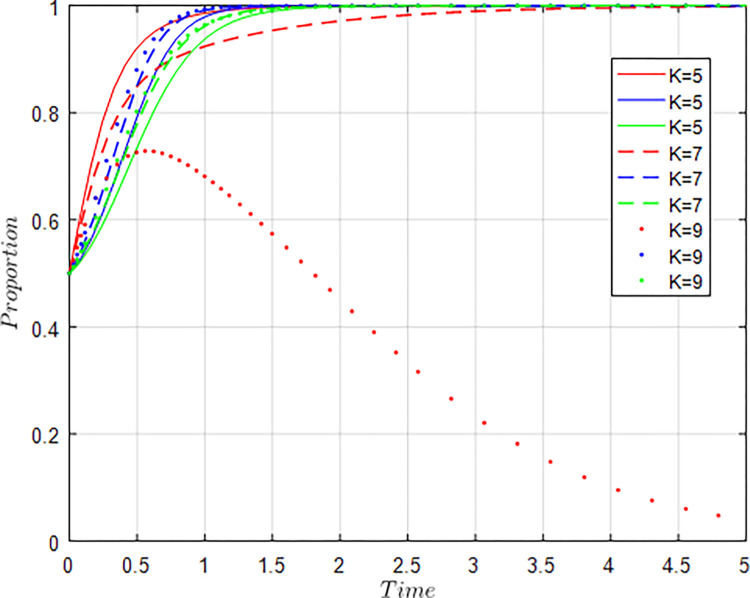
Influence of *K* changes on evolution results.

All other things being equal, let *K* = 5; *K* = 7; *K* = 9 respectively, i.e, other conditions remain unchanged, government incentive cost changes, and the evolution results of equilibrium strategy are shown in Fig 10.

As shown in Fig 10, the threshold value of government incentive cost ranges from 7 to 9. When the government incentive cost is greater than this threshold, the government’s willingness to implement incentive policies will converge to 0, i.e, the government will choose the non-regulatory strategy. Instead, the government will choose a regulatory strategy. Meanwhile, with the increase of government incentive cost, the willingness of agriculture-related enterprises, universities & research institutions to participate in collaborative innovation converges to 1. The simulation results show that the higher the incentive cost of the government, the lower the willingness of the government to implement the regulatory strategy, and the high incentive cost will force the government to give up the regulation. However, within a reasonable range, an appropriate increase in government incentive costs will promote the cooperation of agriculture-related enterprises, universities & research institutions, and contribute to the healthy development of agricultural innovation ecosystem.

(4) The influence of collaborative innovation cost changes in agriculture-related enterprises, universities & research institutions

**Fig 11:**
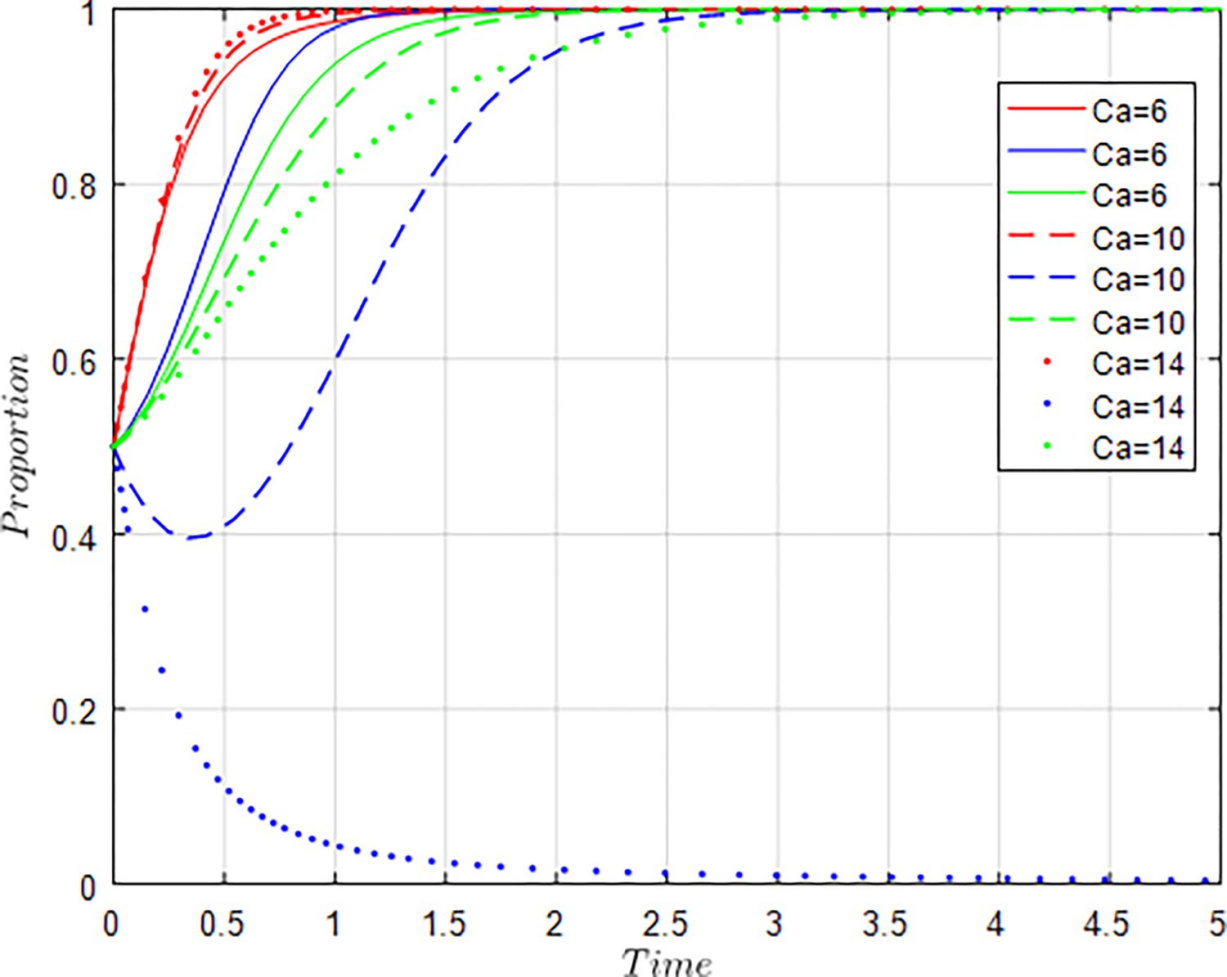
Influence of *C*_*a*_ changes on evolution results.

All other things being equal, let *C*_*a*_ = 6; *C*_*a*_ = 10; *C*_*a*_ =14 respectively,. I.e, other conditions remain unchanged, government regulatory costs change, and the evolution results of equilibrium strategies are shown in Fig 11.

As shown in Fig 11, the threshold value of collaborative innovation cost of agriculture-related enterprises ranges from 10 to 14. When the cost of participating in collaborative innovation exceeds this threshold, the willingness of agriculture-related enterprises to participate in collaborative innovation will converge to 0, i.e., the agriculture-related enterprises will choose non-collaboration strategy. On the contrary, agriculture-related enterprises will choose collaboration strategy. Meanwhile, with the increase of collaborative innovation cost of agriculture-related enterprises, the willingness of universities & research institutions to participate in collaborative innovation is decreasing, but the willingness of the government to implement regulation is increasing. The simulation results show that the higher the cost of collaborative innovation, the lower the willingness to participate in collaborative innovation, and the higher the cost of collaborative innovation will force the enterprises to give up collaboration strategy. In addition, the decrease in the willingness of agricultural enterprises to collaborate on innovation will destroy the stable industry-university-research relationship, thus reducing the willingness of universities & research institutions to collaborate on innovation. In order to maintain the normal operation of the agricultural innovation ecosystem, the government needs to take more active regulatory measures.

**Fig 12:**
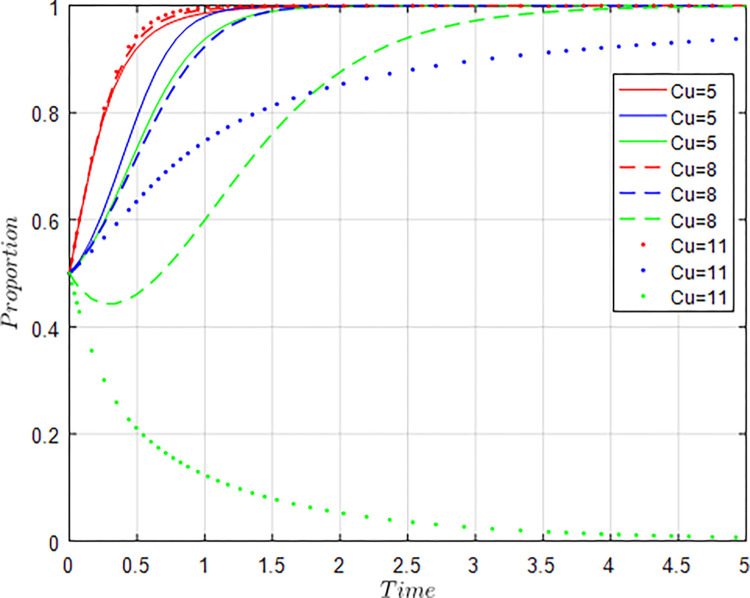
Influence of *C*_*u*_ changes on evolution results.

All other things being equal, let *C*_*u*_ = 5; *C*_*u*_ = 7; *C*_*u*_ = 9 respectively, i.e., other conditions remain unchanged, and the cost of government regulation changes. The evolution results of equilibrium strategy are shown in Fig 12.

As shown in Fig 12, the cost threshold of collaborative innovation between universities & research institutions ranges from 8 to 11. When the cost of participating in collaborative innovation exceeds this threshold, the willingness of universities & research institutions to participate in collaborative innovation will converge to 0, i.e., they will choose non-collaboration strategy. Instead, universities and research institutions will choose collaboration strategy. Meanwhile, the collaborative cost of universities & research institutions will lead to the decrease of agriculture-related enterprises’ willingness to participate in collaborative innovation and the increase of government’s willingness to implement regulation [59]. The simulation results show that the increase of collaborative innovation cost of universities & research institutions will not only affect their own willingness to participate in collaborative innovation, but also reduce the willingness of agriculture-related enterprises because of the difficulty in transforming innovation achievements. This is a time when governments need to play a more active role to keep the agricultural innovation ecosystem running.

As can be seen from the simulation results in Figs 11 and 12, when the collaborative innovation cost of agriculture-related enterprises, universities & research institutions is greater than the threshold value, the will of agriculture-related enterprises, universities & research institutions will converge to 0, and only the will of the government will converge to 1, i.e., the final equilibrium strategy is (regulation, non-collaboration, non-collaboration). In order to promote the tripartite collaborative evolution, it is necessary to increase government support and formulate reasonable reward and punishment measures. This result verifies the conclusion of the stability point analysis of the tripartite evolutionary game.

(5) The influence of changes liquidated damages

**Fig 13:**
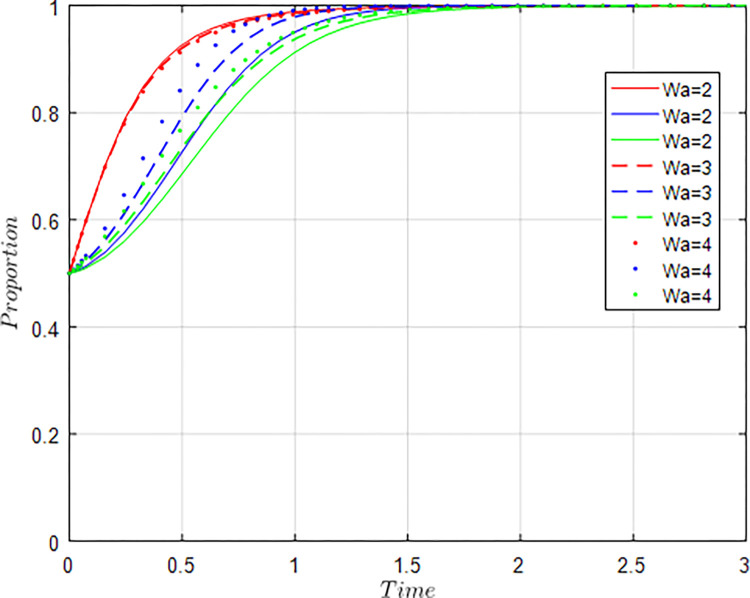
Influence of *W*_*a*_ changes on evolution results.

**Fig 14:**
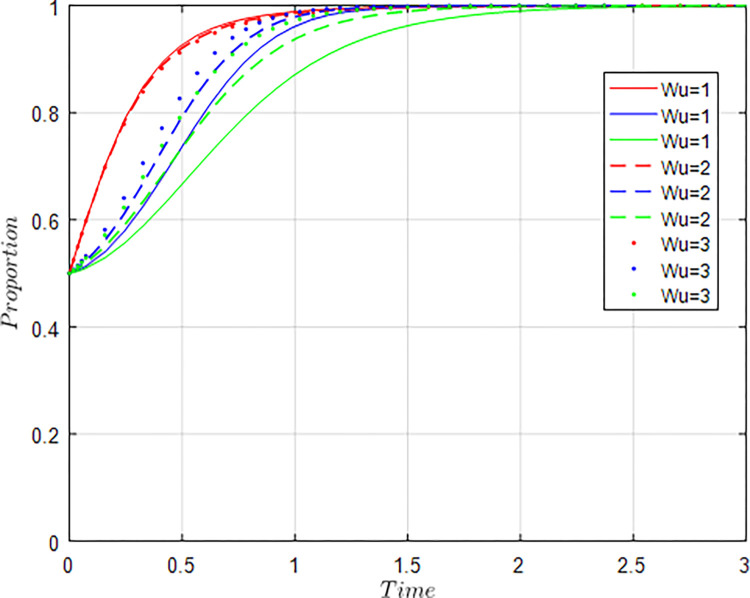
Influence of *W*_*u*_ changes on evolution results.

Assuming *W*_*a*_ = 2; *W*_*a*_ = 3, *W*_*a*_ = 4 and *W*_*u*_ =1; *W*_*u*_ =2; *W*_*u*_ =3 respectively, i.e., the other conditions remain unchanged and the default paid by the agriculture-related enterprises or universities and research institutions betraying collaborative innovation changes, the equilibrium strategy evolution results are shown in Figs 13 and 14.

As shown in Figs 13 and 14, the higher the liquidated damages for the betrayal of collaborative innovation by one of the agriculture-related enterprises or universities & research institutions, the faster the willingness of the other party to participate in collaborative innovation converges to 1, and the speed of converging to 1 for agriculture-related enterprises is higher than the speed of converging to 1 for universities & research institutions. Meanwhile, with the increase of liquidated damages on the part of agriculture-related enterprises or universities & research institutions, the government’s regulatory willingness has shown a downward trend. The simulation results show that the greater the punishment for betraying collaborative innovation, the more collaborative innovation can be promoted, and the punishment for agriculture-related enterprises is more sensitive than that for universities & research institutions. In addition, a strict punishment mechanism can appropriately reduce government regulation.

To sum up, in order to promote the collaborative evolution of agricultural innovation ecosystem, agriculture-related enterprises, universities & research institutions should establish more perfect reward and punishment mechanisms, and cost control mechanisms, then, the government should formulate reasonable regulatory cost and incentive cost ranges to actively guide the atmosphere of collaborative innovation. Especially when the cost of collaborative innovation of agriculture-related enterprises, universities & research institutions is higher than the threshold, it is particularly important to increase government support and formulate strict punishment mechanism for breach of contract.

## 5 Conclusion and enlightenment

### 5.1 Conclusions

Innovation is the primary driving force for agricultural development, and it is also a key element for the modernization of agriculture and rural areas and, ultimately, the revitalization of rural areas. With the promotion of rural collective property rights system reform, land system reform, and supply and marketing cooperative comprehensive reform, the agricultural innovation paradigm has undergone significant changes. Objectively, it is necessary to promote the transformation of the agricultural innovation mode from a more open perspective, break the disadvantages of single industrial chain development, build a diversified agricultural innovation ecosystem, promote multi-body synergistic innovation, and change the transformation from blood transfusion to hematopoietic. Based on the forefront of innovation theory—i.e, the innovation ecosystem research perspective—in this paper, we positioned agriculture as a weak industry. First, the concept of an agricultural innovation ecosystem was defined and its constituent elements and structure were deconstructed. Then, evolutionary game theory was used to construct an evolutionary game model of cooperative innovation involving agricultural-related enterprises, universities, and scientific research institutions under the effects of government rewards and punishment, and numerical simulation was carried out suing MATLAB. Our research conclusions are as follows:

1. The source of innovation in the agricultural innovation ecosystem mainly comes from industry–university–research cooperation, which is at the core of the agricultural innovation ecosystem. Through industry–university–research synergistic innovation, agriculture-related enterprises may acquire mature technologies, advanced knowledge, talents from universities and research institutions, and improved independent innovation capabilities [60]. Meanwhile, universities and research institutions have gained platforms and space for scientific research from agriculture-related enterprises, making scientific research more suitable to the market demand and promoting technological transformation and diffusion.
2. The government, agriculture-related enterprises, universities & research institutions have different degrees of influence on each other’s participation intention. On the one hand, the willingness of agriculture-related enterprises, universities & research institutions to participate in collaborative innovation is mutually promoting. The willingness of one party to participate in collaborative innovation will increase with the willingness of the other party to participate in collaborative innovation. Moreover, the willingness of agriculture-related enterprises to participate in collaborative innovation is more sensitive than that of universities & research institutions. This is because when the willingness of agricultural enterprises or universities & research institutions to collaborate on innovation increases, they will take the initiative to seek industry-university-research cooperation, so as to promote the willingness of the other party to participate in collaborative innovation. Meanwhile, since agricultural enterprises are the direct beneficiaries of innovation results, their willingness to participate is significantly higher than that of universities & research institutions. Therefore, the government should tilt the incentive funds to universities & research institutions, enhance the enthusiasm of universities & research institutions to participate in collaborative innovation, and give full play to the role of innovation. On the other hand, the government’s regulatory willingness will decrease with the increase of the willingness of agricultural enterprises, universities & research institutions to participate in collaborative innovation. This is because at the early stage of the evolution of the agricultural innovation ecosystem, agriculture-related enterprises, universities & research institutions are not willing to participate in collaborative innovation, so the government must play a more active role and encourage agriculture-related enterprises, universities & research institutions to participate in collaborative innovation through policy guidance.
3. The participation cost changes of government, agriculture-related enterprises, universities and research institutions affect the collaborative innovation evolution of agricultural innovation ecosystem. On the one hand, the higher the regulatory cost and incentive cost, the lower the regulatory meaning. When the regulatory cost and incentive cost are too high, the government will withdraw due to excessive financial burden. Meanwhile, changes in government supervision cost and incentive cost have opposite effects on the willingness of agriculture-related enterprises, universities & research institutions to participate in collaborative innovation. With the increase of government supervision cost, the willingness of agriculture-related enterprises, universities & research institutions will decline; with the increase of government incentive cost, the willingness of agriculture-related enterprises, universities & research institutions will also increase. This is because excessive government intervention will affect the normal operation of the market mechanism, and then affect the cooperation between enterprises, universities and research institutes. The increase of incentive cost will mobilize the enthusiasm of agriculture-related enterprises, universities & research institutions to participate in collaborative innovation. Therefore, the government should establish a reasonable range of regulatory costs and incentive costs to ensure the smooth development of collaborative innovation activities. On the other hand, the cost of agricultural enterprises, universities & research institutions participating in collaborative innovation will also affect each other’s willingness to participate. The higher the cost of collaborative innovation, the lower the willingness of agricultural enterprises, universities & research institutions to participate in collaborative innovation. Therefore, a more complete cost control mechanism should be established to maintain the operation of agricultural innovation ecosystem.
4. The changes of liquidated damages affect the willingness of agriculture-related enterprises, universities and scientific research institutions to participate in collaborative innovation. The higher the liquidated damages set, the more it can promote collaborative innovation, and the punishment of agriculture-related enterprises for betraying collaborative innovation is more sensitive than that of universities and scientific research institutions. However, when the liquidated damages are set too high, it may cause agriculture-related enterprises, universities and research institutions to fear high penalties and choose to conduct R&D alone. Therefore, an effective liquidated damages system and a reasonable amount of liquidated damages should be formulated to create a favorable environment for collaborative innovation.

### 5.2 Contribution

Based on the practice of Chinese agricultural industry development, this paper explores key issues related to agricultural innovation ecosystems and their evolutionary processes from an innovation ecosystem perspective, and the main research contributions are summarized as follows:

1. Early research on innovation ecosystems mostly involved high-tech industries, strategic emerging industries, cultural and creative industries and other industries with high technological content, and the scope of research was mostly in developed regions of developed countries. As the research progresses, the relevant theoretical research gradually begins to be refined, but the relevant research is still very limited for the low technology content industries in developing countries, especially the weak agriculture. Based on the current situation of China’s agricultural industry, this paper proposes the connotation of agricultural innovation ecosystem and constructs an integrated framework to deconstruct agricultural innovation ecosystem, which breaks through the traditional "point-to-point" linear innovation paradigm of agriculture and conducts a more systematic, ecological and organic research for the coordinated development of economy, society and ecology, which is an important contribution to enrich and expand the innovation ecosystem theory.
2. Although the theory of innovation ecosystem has been relatively mature and the number of related research results has increased year by year, the research on agricultural innovation ecosystem has just started, and it still remains on some superficial research of theoretical framework construction, and lacks the internal logic and mechanism analysis under the guidance of specific system thinking. Moreover, most existing studies analyze agricultural innovation ecosystems from a static perspective, and do not reveal the evolutionary relationship among innovation agents from a dynamic perspective. In addition, the existing research results on evolutionary game mainly focus on the two-party game between the core firm and the complementary firm, and the multi-party evolutionary game research is relatively rare. To break through the plight of agricultural independent innovation ability promotion, production, study and research, this paper establishes a tripartite collaborative innovation evolution game model of government, agriculture-related enterprises, universities and scientific research institutions, and reveals the dynamic evolution process of collaborative innovation in agricultural innovation ecosystem through numerical simulation. The relevant research in order to promote agricultural innovation ecosystem value to create a collaborative innovation provides a decision-making reference.
3. Considering the limitation of agricultural innovation resources in developing countries, this paper takes China, the largest developing country, as the research object to study the agricultural innovation ecosystem and its evolution. The relevant results can break through the dilemma of low-end locking of agricultural science and technology innovation in developing countries, and promote intra- and inter-regional synergistic and integrated innovation of multiple subjects from the perspective of open innovation, so as to realize a convergent development path of symbiosis, sharing and co-creation, and contribute to high-quality agricultural development and rural revitalization in developing countries.

### 5.3 Implications

This paper is oriented to developing countries, based on the research perspective of innovation ecosystem, locates agriculture as a weak industry, and studies the evolution process of agricultural innovation ecosystem and its collaborative innovation based on the development status of Chinese agricultural industry. The present research results explore and extend the theoretical and fundamental research on agricultural innovation ecosystems, providing a better exploration of innovation-driven development in agriculture. In order to better maintain the stability of the agricultural innovation ecosystem and promote the sustainable development of agriculture, we put forward the following suggestions:

1. The goal of cooperation should be unified and fully communicated, in order to ensure cooperation between the industry, universities, and research institutes has a common direction and sustainability, in order to avoid the failure of cooperation due to inconsistent goals.
2. A mechanism for sharing interests and risks should be established, in order to clarify the responsibilities of all parties and choose different modes of benefit distribution according to the different modes of cooperation. For example, risk and return may be linked to the investment proportion. Under the premise of clear property rights, a diversified profit distribution mechanism can be stimulated by way of joint construction, technological investment, and equity incentive. Furthermore, third-player institutions can be introduced, a multi-player conflict coordination mechanism can be established, and the formation of a risk-sharing mechanism can be promoted.
3. Give full play to the role of the government, in terms of establishing effective reward and punishment mechanisms, guiding and coordinating the members of all parties, establishing agricultural innovation projects, building platforms to attract agriculture-related enterprises and research institutions to participate in agricultural science and technology innovation activities, and the establishment of multiple financing channels, in order to provide favorable financial support for industry–university–research synergistic innovation.
4. The government should play a role in decision making, while reducing direct intervention in agricultural science and technology innovation activities, giving full play to the decisive role of the market, allocating more innovation resources to the dynamic and potential agriculture-related enterprises, and cultivation agriculture-related enterprises to become the core subject of the regional agricultural innovation ecosystem.

### 5.4 Deficiencies and future prospects

The main deficiency of this paper is that, when studying the collaborative innovation evolution process of agricultural innovation ecosystems, universities & research institutions were regarded as the parties for the analysis. In fact, there are partial differences between the functions of universities & research institutions in synergistic innovation, and this paper does not distinguish between them. In addition, in the numerical simulation of the collaborative innovation evolution of agricultural innovation ecosystem, the valuation technology was used for simulation without using actual research data, which is relatively lacking in guiding significant practices. Therefore, future research should continue to follow-up on these results and, on the basis of distinguishing the functions of universities and research institutions, further study the evolutionary game process of cooperative innovation in agricultural innovation ecosystems, as well as collecting research data for simulation, such that the analysis results may be more scientific.
